# IL-17A and TNF-α inhibitors induce multiple molecular changes in psoriasis

**DOI:** 10.3389/fimmu.2022.1015182

**Published:** 2022-11-22

**Authors:** Qiang Dong, Dan Li, Bi Bo Xie, Li Hua Hu, Jia Huang, Xiao Xiao Jia, Yan Li Tang, Gan Hong Liu, Ning Ning Shen, Xiao Bing Yu

**Affiliations:** Department of Dermatology, Dermatology Hospital of Zhejiang Province, Huzhou, Zhejiang, China

**Keywords:** proteomics, psoriasis, parallel reaction monitoring, data-independent acquisition mass spectrometry, biological agent, ingenuity pathway analysis, adalimumab, secukinumab

## Abstract

Adalimumab and secukinumab are commonly used for moderate to severe psoriasis vulgaris (PV). Although distinct individual responses to and impaired effectiveness of these biological agents occur occasionally, little is known about the underlying reasons. Here, we report a proteomic analysis of psoriatic lesions from patients treated with these drugs using data-independent acquisition mass spectrometry (DIA-MS). Thousands of differentially expressed proteins (DEPs) changed over 12 weeks of treatment. Network analysis showed that DEPs could interact and induce transformation in matrix components, metabolic regulation, and immune response. The results of parallel reaction monitoring (PRM) analysis suggested that S100s, STAT1, KRT2, TYMP, SOD2, HSP90AB1, TFRC, and COL5A1 were the most significantly changed proteins in both groups. There was a positive association between the Psoriasis Area and Severity Index (PASI) score and three proteins (TFRC, IMPDH2, KRT2). Our study findings suggest that inhibition of IL-17A and TNF-α can induce changes in multiple molecules in psoriatic lesions and have an overlapping influence on the immune response and process through direct or indirect effects.

## Introduction

PV can cause multisystem damage as well as skin damage. Research has highlighted the vital roles of adaptive immune molecules, such as IL-17, IL-23, and TNF-α (1), in PV, which has allowed the development of multiple effective therapies targeting tumour necrosis factor-alpha (TNF-α; adalimumab), IL-17A (secukinumab), IL-17 receptor A (IL-17RA; brodalumab), and IL-23 (guselkumab) (2–4). Among them, secukinumab and adalimumab are the two most widely used biological agents in China. These biological agents have significantly improved the efficacy of psoriasis treatment (5), but a loss of effectiveness (6) or the occurrence of phenotypic transformation (7) is not uncommon in clinical practice. These phenomena suggested that using biological agents might alter immune pathology and highlight the pleiotropic nature of these molecules. Further investigations are needed to explain these phenomena.

Recent developments in the field have moved towards delivering high-quality and consistent quantification in large-scale projects. DIA-MS is emerging as a technology that combines deep proteome coverage capabilities with quantitative consistency and accuracy (8). DIA-MS has been successfully used for the investigation of skin tumours (9) and atopic dermatitis (AD) (10). Meng et al. used DIA-MS to identify serum proteins distinguishing responders from nonresponders to an oral traditional Chinese medicine to explore diagnostic and predictive disease biomarkers in serum (11), and they demonstrated the clinical utility of an in-depth serum proteomic platform to identify specific diagnostic and predictive biomarkers of psoriasis. These results suggest that DIA-MS can be used to study the skin of patients with psoriasis.

A few studies have provided significant insights into the changes in proteins or genes in psoriatic lesions after treatment with biological agents. Kolbinger et al. quantified the levels of 170 proteins in patients with psoriasis before and after administration of the secukinumab. The results showed that many dysregulated immune molecules returned to normal after treatment with secukinumab (12). Foulkes et al. investigated the serum proteome of patients with severe psoriasis treated with the TNF inhibitor etanercept. The study found that the baseline serum proteome can indicate patient response to biological therapies (13). However, the platforms used in these previous studies have limited ability to detect protein species. Therefore, the characterization of the change in proteins in the skin after treatment with biological agents through DIA-MS is an attractive possibility.

Here, we used DIA-MS to delineate the proteomic landscape of the skin of patients with moderate to severe psoriasis. In summary, from a posttreatment point of view, these data identified IL-17A and TNF-α as critical molecules that maintain psoriasis plaques. The inhibition of these molecules can induce massive protein changes over time and have overlapping influences on immune response and process through direct or indirect effects.

## Materials and methods

### Patients and samples

This study enrolled patients from a two-part, prospective cohort study that included patients with moderate to severe psoriasis. Patients were 18 years of age or older. Patients in the three groups were included if they had a minimum treatment duration of three months with biological agents, immunosuppressants or phototherapy. We excluded patients who has contraindications to biological agents, or has other inflammatory skin diseases, such as eczema. Patients had inadequately controlled psoriasis for more than six months (see **Table 1**). All patients in the biological agent group were treated according to the clinical standard dose. The patients in the control group were administered drugs for local medications, drugs other than systematic immunosuppressants or combined with phototherapy. Participants were assessed and sampled at baseline, week 1, week 4, and week 12 from April 2020 to February 2022 in the Dermatology Hospital of Zhejiang Province. All patients were diagnosed with moderate to severe psoriasis (BSA≥10% or PASI≥12). The response to therapy was assessed using the Psoriasis Area and Severity Index (PASI). The study was approved by ethical committees at the Dermatology Hospital of Zhejiang Province and performed according to the guidelines of the Declaration of Helsinki. Patients provided written informed consent before sample collection. We used a biopsy punch with a diameter of 4 mm for sampling lesional (LS) plaques. The skin specimens were placed in 5-mL Eppendorf tubes, immediately frozen in liquid nitrogen for 5-10 min, and stored at -80°C.

**Table 1:** Baseline characteristics of psoriasis patients.

	Test research			Validation research		
Variables	Adalimumab	Secukinumab	Controls	Adalimumab	Secukinumab	Controls
Sex- no. (%)						
Male	3 (75.0)	4 (100.0)	3 (75.0)	3 (60.0)	3 (60.0)	5 (100.0)
Female	1 (25.0)	0 (0.0)	1 (25.0)	2 (40.0)	2 (40.0)	0 (0.0)
Age, year						
Mean ± SD.	41.5 ± 15.3	51.8 ± 21.6	55.8 ± 10.2	38.2 ± 8.0	47.0 ± 24.5	45.2 ± 4.3
Median (IQR)	37.5 (29.5-57.5)	49.0 (33.3-73.0)	55.0 (47.0-65.3)	39.0 (31.0-45.0)	32.0 (28.0-73.5)	48.0 (40.5-48.5)
Range	28.0-63.0	33.0-76.0	47.0-66.0	26.0-47.0	27.0-78.0	40.0-49.0
Course, year						
Mean ± SD.	11.8 ± 9.0	23.0 ± 18.0	27.8 ± 12.2	11.4 ± 8.2	13.0 ± 16.8	12.2 ± 11.8
Median (IQR)	8.0 (6.0-21.3)	14.5 (13.3-41.3)	22.0 (21.3-40.0)	10.0 (5.0-18.5)	6.0 (5.0-24.5)	5.0 (3.0-25.0)
Range	6.0-25.0	13.0-50.0	21.0-46.0	4.0-25.0	5.0-43.0	3.0-27.0
Previous treatment - no. (%)						
Topical therapy	4 (100.0)	4 (100.0)	3 (75.0)	4 (80.0)	5 (100.0)	5 (100.0)
Systemic therapy	4 (100.0)	3 (75.0)	4 (100.0)	4 (80.0)	5 (100.0)	5 (100.0)
Phototherapy	3 (75.0)	2 (50.0)	1 (25.0)	3 (60.0)	5 (100.0)	2 (40.0)
Biologic therapy	2 (50.0)	1 (25.0)	0 (0.0)	0 (0.0)	1 (20.0)	0 (0.0)
Family history - no. (%)						
Yes	1 (25.0)	1 (25.0)	2 (50.0)	1 (20.0)	3 (60.0)	0 (0.0)
No	3 (75.0)	3 (75.0)	2 (50.0)	4 (80.0)	2 (40.0)	5 (100.0)
PASI (0 weeks), score						
Mean ± SD.	20.7 ± 8.1	20.4 ± 9.3	17.1 ± 7.7	25.3 ± 9.4	22.4 ± 2.8	19.5 ± 6.6
Median (IQR)	19.6 (13.8-28.7)	18.0 (13.0-30.2)	16.2 (10.4-24.8)	20.4 (18.0-35.1)	23.4 (20.0-24.3)	18.0 (13.5-26.3)
Range	12.0-31.5	12.3-33.3	10.0-26.0	18.0-39.0	17.6-24.6	12.2-27.8
PASI (1 week), score						
Mean ± SD.	15.6 ± 10	14.6 ± 6.2	13.4 ± 7.9	21.8 ± 8.5	17.7 ± 3.5	15.5 ± 7.0
Median (IQR)	12.8 (8.0-25.8)	12.3 (10.2-21.2)	10.4 (8.1-21.8)	18.0 (15.9-29.7)	19.5 (14.8-19.7)	12.0 (9.7-23.0)
Range	6.8-29.8	10.2-23.4	8.1-24.8	15.6-36.0	11.6-19.8	8.4-24.0
PASI (4 weeks), score						
Mean ± SD.	7.4 ± 8.9	7.9 ± 3.2	9.4 ± 6.8	15.0 ± 4.5	12.2 ± 2.5	10.4 ± 4.9
Median (IQR)	3.4 (2.4-16.5)	9.2 (4.6-9.9)	9.2 (3.1-15.9)	12.3 (11.7-19.6)	13.2 (9.9-14.0)	9.0 (6.3-15.2)
Range	2.1-20.7	3.1-10.1	2.4-16.8	11.4-21.9	8.0-14.4	5.8-17.8
PASI (12 weeks), score						
Mean ± SD.	3.2 ± 2.7	1.75 ± 0.9	14 ± 15.1	9.1 ± 3.0	6.6 ± 2.5	7.3 ± 5.7
Median (IQR)	1.7 (1.6-.)	1.75 (0.9-2.6)	9.3 (3.2-29.6)	8.7 (6.4-12.2)	7.0 (4.4-8.8)	5.9 (2.7-12.6)
Range	1.6-6.3	0.8-2.7	1.5-36.0	6.0-13.3	3.2-9.8	2.0-16.4

no. (%), number; SD, standard deviation; IQR, interquartile range.

### Proteomic data acquisition

#### DIA analysis

After sample preparation and liquid nitrogen grinding+SDT lysis, 1 g of peptides was collected from each sample, iRT peptides mixed lagging samples, separated by nano-LC and analysed by online electrospray tandem mass spectrometry. The whole liquid mass series system was as follows: 1) liquid phase system: Easy nLC system (Thermo Fisher Scientific); and 2) mass spectrometry system: Orbitrap Exploris 480 (Thermo Fisher Scientific).

#### DIA data analysis

Data from DIA were processed and analysed by Spectronaut 14.6 (Biognosys AG, Switzerland) with default settings, and the retention time prediction type was set to dynamic iRT. Data extraction was determined by Spectronaut X based on extensive mass calibration. Spectronaut 14.6 will dynamically determine the ideal extraction window depending on iRT calibration and gradient stability. The Q value (FDR) cut-off at the precursor and protein levels was 1%. Decoy generation was set to mutate, similar to scrambled but applying only a random number of AA position swamps (min=2, max=length/2). All selected precursors passing the filters were used for quantification. MS2 interference removes all interfering fragment ions except the three least interfering ones. The average top 3 filtered peptides that passed the 1% Q value cut-off were used to calculate the significant group quantities.

#### Quality control of proteome data

DIA analysis of 47 samples was performed using the database constructed by deep DIA. To ensure the accuracy of the quantification for the results obtained, we first normalized the DIA results, which excluded systematic errors to some extent **(** **Figure S1** **)**. The CV value interval statistics and sample consistency assessments were then performed. The quality of proteomic data was ensured at multiple levels.

#### PRM

Each 1-g sample was fed into the system, separated by nano-LC, and analysed by online electrospray tandem mass spectrometry. Mass spectrometry parameters were set as follows: (1) full-MS: scan range (m/z)=350-1800, resolution=60,000, AGC target=3e6, and maximum injection time=50 ms; and (2) PRM: resolution=30,000, AGC target=2e5, maximum injection time=50 ms, loop count=10, isolation window=2.0 m/z, and NCE=27 eV.

#### Bioinformatic analysis

A heatmap was drawn using the pheatmap package and RColorBrewer package in the R language (version 4.0.1). The molecular function of DEPs was analysed using the GO database and visualized by Cytoscape (14). Pearson correlation was used to represent correlations between DEPs and clinical improvement, measured by the PASI, and a circus plot was made using the circlize package in the R language (version 4.0.1) (15). The pathway enrichment and canonical network analysis were analysed by IPA (Version 9.0, Ingenuity Systems, Redwood City, CA, USA) (with p value < 0.05, Z score > 0 or < 0) (16).

#### Statistical analysis

Student’s t test was performed to compare quantitative data between the two groups. Log_2_ fold-change (FC) was calculated by the mean protein expression ratio between the biological agent and controls. The selection criteria of the DEPs for bioinformatic analysis were that the p value should be less than 0.05 and |log _2_(FC)| should be larger than log_2_ (1.2). Statistical analysis was performed using R (version 4.0.1).

## Results

### Generation and characterization of the proteomic landscape

Forty-seven samples were collected from twelve patients with PV in the DIA research. Then, PRM validation was performed in validation research. Detailed demographic and clinical information, including treatment history, sex, age, disease course, family history, and PASI score at 0, 1, 4, and 12 weeks, are summarized in **Table 1**. We quantified 6054 proteins with a false discovery rate (FDR) of less than 1% at both the peptide and protein levels. We analysed four technical replicates of randomly selected tissue samples and four pooled controls for each DIA batch. Protein quantification in these technical replicates and control samples showed a relatively low median coefficient variance (CV) of <20% and <10%, respectively (**Figure S2**).

**Figure 1:**
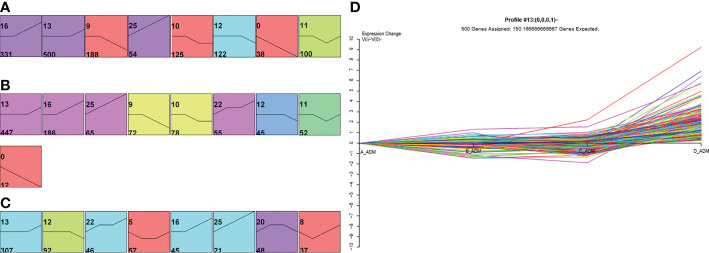
Expression patterns of differentially expressed proteins (DEPs) in the three groups. Expression patterns of DEPs in the adalimumab group **(A)**, secukinumab group **(B)**, and control group **(C)**. Coloured models enriched a statistically significant number of proteins. The number on the top left corner indicates the serial number of models, and the bottom left corner reveals the number of proteins enriched. **(D)** Representative protein expression pattern of Model 13 (**A**–**D** represent baseline, week 1, week 4, and week 12, respectively) in the adalimumab group.

First, to investigate the expression profiling of proteins throughout different treatment times, we used Short Time-series Expression Miner (STEM) to depict the dynamic expression patterns according to a previous study (17). A total of 1458 and 1012 proteins were significantly changed in the adalimumab and secukinumab groups compared to controls (653). The numbers of DEPs in the biological group were considerably higher than those in the control group. A total of 885 and 753 proteins were upregulated in the adalimumab and secukinumab groups, respectively. The adalimumab group enriched eight models (**Figure 1A**), and the secukinumab group enriched nine models (**Figure 1B**).

### More DEPs begin to normalize in the early stage

We next distinguished DEPs in psoriatic skin at the early (1 week) and late (12 weeks) stages of psoriasis treated with biological agents, setting the fold change (FC) to be two or greater or -2 or less (FC> ± 2) using STEM. Finally, 268 and 158 proteins in the adalimumab group and secukinumab group were analysed, respectively. It was found that more DEPs began to normalize in the early stage than in the late stage in both groups (**Table S1**). Notably, MX1, KRT16, S100A8, and S100A9 were significantly reduced at an early stage in both groups.

### Six enriched molecular functions in GO analysis

**Figure 2:**
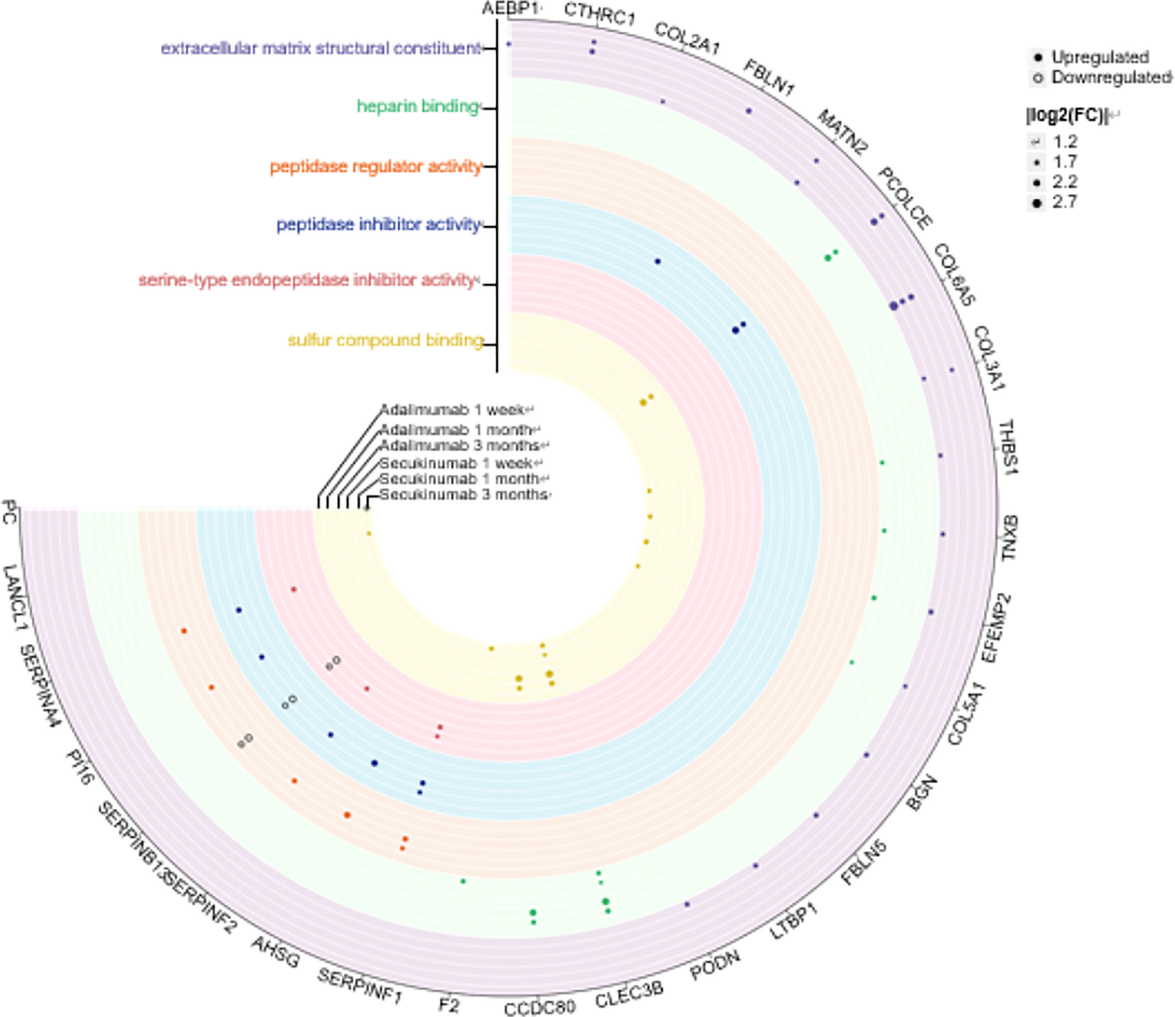
Six clusters of molecular functions in the two biological agent groups according to the GO enrichment analysis. The DEPs are labelled with circles (solid, upregulated proteins; hollow, downregulated proteins). Different colours represent different clusters. The size of the circle indicates |log_2_ (FC)|. The cut-off was set at p value<0.05 and |log_2_ (FC)| >log_2_ (1.2).

Next, we depicted a Venn diagram for screening DEPs to rule out confounding effects from baseline protein differences. Finally, 216 and 178 proteins were identified separately in the adalimumab and secukinumab groups (**Figure S3**). We performed GO enrichment analysis to investigate the molecular functions of these proteins. Six significantly enriched GO terms are displayed in **Figure 2**. Notably, many DEPs were enriched in extracellular matrix structural constituents, which are crucial in cutaneous homeostasis and repair, but disorders occur in inflammatory skin diseases such as psoriasis. We also found several proteins belonging to the “SERPIN family”, particularly in the adalimumab group.

### Correlation analyses between DEPs and PASI scores

Remarkable clinical improvement after treatment with these two biological agents was achieved. There was a mean percentage reduction in the PASI score of 73.6% (p<0.001) in the adalimumab group and 79.2% (p<0.0001) in the secukinumab group, compared to 44.3% in controls (p>0.05) at week 12. There was no significant difference in PASI scores after 12 weeks among the groups (**Figure S4**), which might be caused by the small sample size. After four weeks, clinical improvement continued in the secukinumab group but not in the adalimumab group.

**Figure 3:**
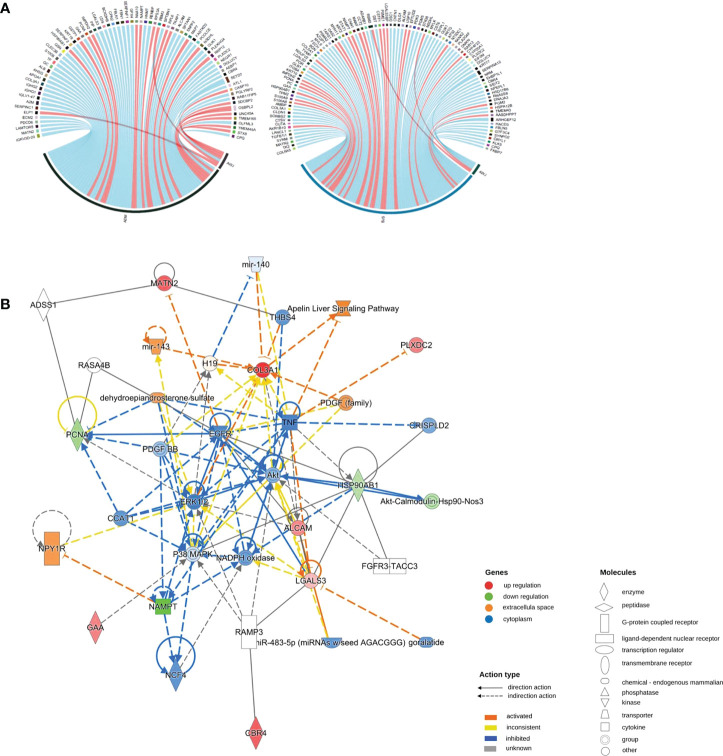
**(A)** Correlation between DEPs and clinical improvement. Circos plot presenting the Pearson correlation between DEPs and the PASI score (|cor|>0.6, p value<0.05). Red ribbons indicate positive Pearson correlation coefficients, namely, the downregulation of protein. Blue represents negative Pearson correlation coefficients, that is, the upregulation of protein. The width of the ribbons indicates the correlation value. **(B)** Causal network analysis obtained by IPA of eleven proteins that changed significantly in both biological agent groups in DIA. The highest-score network (containing ten proteins, except IMPDH2) is displayed only for the secukinumab group because the relationship patterns of the proteins in the two groups were similar. The relationship among molecules is represented by lines (solid lines for direct association and dotted lines for indirect association).

We performed Pearson correlation analyses to explore the associations between DEPs and PASI scores. The positive and negative correlation data are presented in **Figure 3A**. In the adalimumab group, the five proteins with the highest positive Pearson correlation coefficients(p-PCCs) were TMEM165, OLFML3, TMEM45A, STX8 and CPQ. In contrast, the five proteins with the highest negative Pearson correlation coefficients (n-PCCs) were IGKV3D-20, MATN2, LAMTOR5, PDCD6 and ECM2. In the secukinumab group, the five proteins with the highest p-PCCs were PGM2, OAS2, AKR1B10, NAMPT and MANF, while the five proteins with the highest n-PCCs were PDK3, ALCAM, SERPINA12, PGM5 and MYH11.

Eleven proteins, including PLXDC2, IMPDH2, ALCAM, HSP90AB1, GAA, COL3A1, PCNA, LGALS3, MATN2, NAMPT, and CBR4, changed significantly in both biological agent groups and had a similar variation tendency. We further analysed these proteins using causal network analysis obtained from ingenuity pathway analysis (IPA) to explore their relationships and possible biological effects. The results showed that molecules in the score network (score 29) were relevant to top diseases and functions such as the cell cycle, cell-to-cell signalling and interaction, and organismal injury and abnormalities. Through complicated direct or indirect connections, some of these molecules affected important immune pathways, such as AKT, TNF, EGFR, ERK1/2, and P38 MAPK, which also form complex relationship networks (**Figure 3B**).

### Overlapping and separating pathways in psoriatic skin after biological treatment

**Figure 4:**
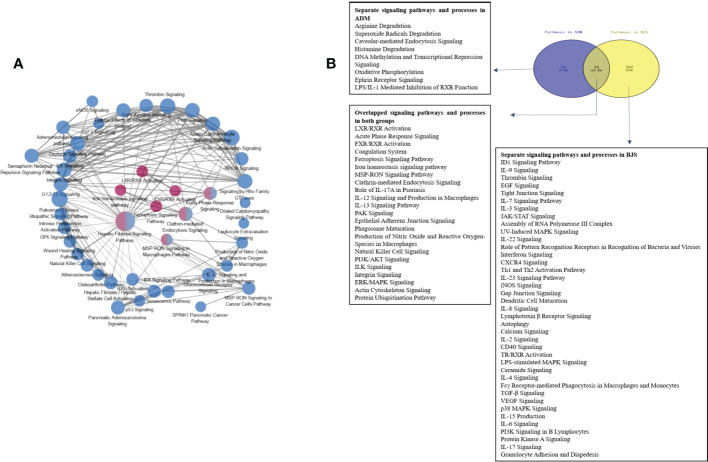
**(A)** Networks of enriched pathways. Nodes represent different pathways, and edges represent interrelation between pathways. Edge width corresponds to the score of a specific pathway pair. The degree of a pathway was represented by node size. The red nodes represent the enriched pathway in the adalimumab group. The blue nodes are enriched in the secukinumab group. **(B)** Separate and overlapped pathways and processes in the two biological agent groups. A Venn diagram was used to screen the skin-related signalling pathways and processes obtained from IPA. The term “ADM” represents adalimumab, while “BJS” represents secukinumab in the picture.

According to the Z score, the significantly altered pathways are illustrated in **Figure S5**. Interestingly, we did not detect changes in the TNF-α or IL-17 pathway. Then, we performed pathway crosstalk analysis to explore the interrelationships among the enriched pathways using the methods of a previous study (18). We found that networks were mainly involved in three major biological processes. The first was metabolism-related pathways, mainly nuclear receptor liver X receptor (LXR)/RAR and farnesoid X receptor (FXR)/RXR activation. The second type was innate immune system-related pathways, such as MSP-RON signalling in the macrophage pathway. The third was adaptive immunity-related pathways, for example, IL-12 signalling and production in macrophages (**Figure 4A**).

Interestingly, we found that the separate pathways in the adalimumab group were mainly related to metabolism, while in secukinumab group, they primarily focused on the immune system. The overlapping part in both groups was related to metabolic and immune pathways and processes (**Figure 4B**).

### Networks of DEPs in nine significantly enriched pathways

**Figure 5:**
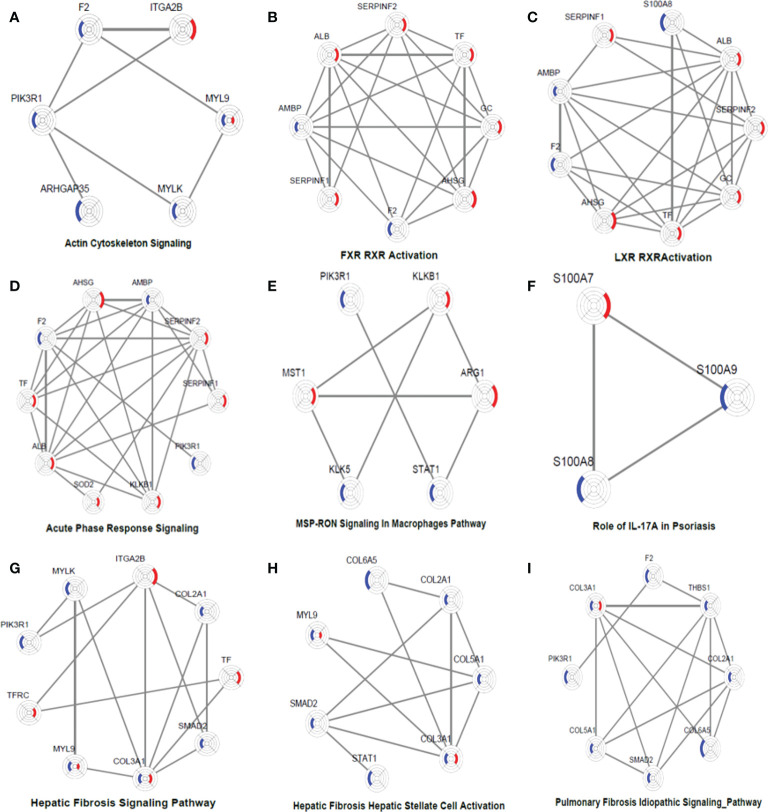
DEP networks in nine significantly enriched pathways. They are actin cytoskeleton signalling **(A)**, FXR_RXR activation **(B)**, LXR_RXR activation **(C)**, acute phase response signalling **(D)**, MSP-RON signalling in macrophages pathway **(E)**, Role of IL-17A in psoriasis **(F)**, hepatic fibrosis signalling Pathway **(G)**, hepatic fibrosis_ hepatic stellate cell activation **(H)**, and pulmonary fibrosis idiopathic signalling pathway **(I)**. Each protein is depicted as a radar chart. Different groups are labelled with different colours. Red area represents the adalimumab group, and blue area represents the secukinumab group. The shadow area covering the circles indicates the FC values for each protein.

To obtain a systematic understanding of the synergetic networks of DEPs in these pathways, we performed network analysis for nine selected significantly enriched pathways using IPA (absolute [Z score] ≥1) between the two groups. We found multiple proteins in the actin cytoskeleton pathway that were altered after biological agent treatment, such as MYLK, F2, and ITGA2B (**Figure 5A**). A few DEPs, such as SERPINC1, SERPINF2, F2, PLXDC2, and TYMP, were also involved in regulating blood coagulation and angiogenesis, which plays an essential role in the pathogenesis and maintenance of psoriasis.

In the adalimumab group, some proteins, such as ALB, TF, GC, SERPINF1, SERPINF2, and AHSG, were involved in the LXR/RAR activation pathway, which is consistent with the findings of previous studies. These metabolic pathways were not activated in the secukinumab group, although some proteins were also involved, such as F2 and AMBP (**Figures 5B–C**). These proteins are also involved in the acute-phase response, which is a core of the innate immune response (**Figure 5D**).

Some essential proteins were involved in immune response pathways. For instance, signal transducer and activator of transcription 1 (STAT1) and arginase 1 (ARG1) participated in the macrophage stimulating protein (MSP)–recepteur d’origine nantais (RON) signalling pathway (**Figure 5E**). In our study, S100A7, S100A8 and S100A9 were significantly involved in the IL-17A pathway, which is strongly implicated in psoriasis pathogenesis (**Figure 5F**).

### F2 activated SMAD2 and MYLK

**Figure 6:**
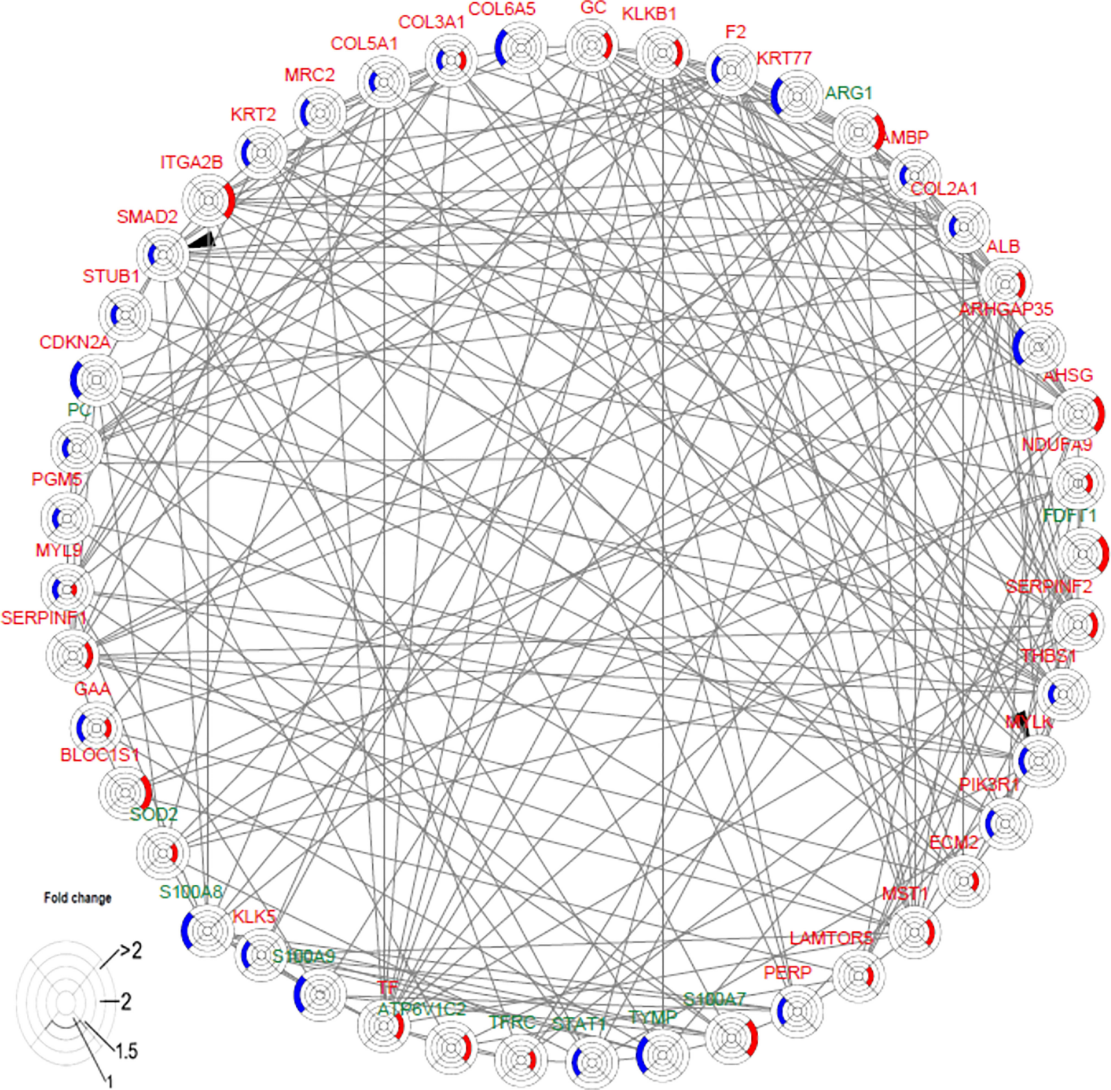
Causal network analysis obtained from ingenuity pathway analysis. Each protein is depicted with a radar chart that is made up of four rings; from the inside to out, it represents |log_2_FC|<=1, |log_2_FC|<=1.5, |log_2_FC|<=2, and |log_2_FC|>2. Red area represents the adalimumab group, and blue area represents the secukinumab group. The relation between two proteins is shown in lines (the arrow indicates predictive activation). Green colour for protein names indicates downregulation, and red colour indicates upregulation. Then, we applied Cytoscape (Version 3.9.1) to visualize the networks. The cut-off was set at p value<0.05 and |log 2(FC)| >log_2_(1.2).

The interaction network among DEPs was constructed using IPA in the two groups (**Figure 6**). We found that F2 can activate SMAD2 and MYLK according to the prediction. Prothrombin (F2) was upregulated in the secukinumab group and involved in actin cytoskeleton signalling, acute phase response signalling, and metabolic regulatory pathways (**Figures 5A–D**). Mothers against decapentaplegic homolog 2 (SMAD2) are transcriptional modulators activated by transforming growth factor-beta (TGF-β). SMAD2 was upregulated, which means that TGF-β receptors increased after treatment since TGF-β receptors were significantly reduced in the psoriatic epidermis. This result suggested that elevated TGF-β/Smad signalling attenuates keratinocyte proliferation while stimulating fibrosis formation (**Figures 5G–I**).

### PRM for protein validation

**Figure 7:**
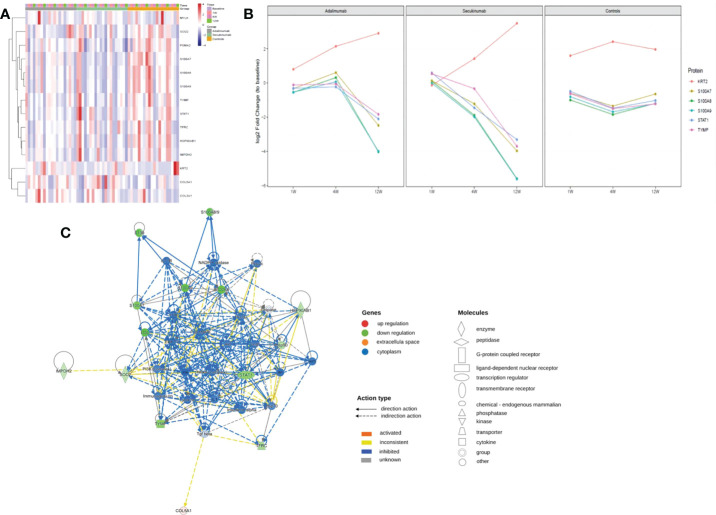
PRM identifies dynamic changes in DEPs during treatment. **(A)** Heatmap shows dynamic changes in fourteen proteins at the different time points and in diverse groups based on the proteomic data of PRM. **(B)** Six proteins with the most significant change in both biological groups and their expression trends at different times. The ordinate represents the log_2_ FC values. **(C)** Causal network analysis obtained by IPA of eleven proteins that changed significantly in both biological agent groups in PRM. The highest-score network (containing ten proteins, except KRT2) is displayed only for the secukinumab group because the relationship patterns of the proteins in the two groups were similar. The relationship among molecules is represented by lines (solid lines for direct association and dotted lines for indirect association).

We screened thirty proteins for further validation using PRM in a separate cohort study of samples. Ultimately, twenty-seven proteins were quantitated, and fourteen proteins were significantly changed at the endpoint of treatment (**Figure 7A**). Except for MYLK in the secukinumab group, which did not change significantly and the results were inconsistent with the results of proteomic analysis, the expression trends of the remaining DEPs were consistent with the microarray analysis. The PRM results verified the reliability of DIA proteomics. S100A7, S100A8, S100A9, STAT1, KRT2, TYMP, SOD2, HSP90AB1, TFRC, and COL5A1 were the most significantly changed proteins in both groups. The trend in the six proteins with the most significant changes over time is shown in **Figure 7B**. Although S100s, STAT1, and KRT2 were significantly different at baseline, which may be attributed to disease severity, there were significant changes among them during biotherapy and no significant changes in the controls. There was a positive association between the PASI score and three proteins (TFRC, IMPDH2, KRT2) in both groups (**Table S2**).

Further IPA network analysis revealed that molecules in the score network (score 29) were relevant to dermatological diseases and conditions, immunological disease, and inflammatory disease. Although most are downstream effector molecules, some of them can affect important pathways, such as the PI3K-AKT, ERK, NF-κB (complex), IL-17R, MAPK, and interferon-alpha signalling pathways (**Figure 7C**).

## Discussion

**Figure 8:**
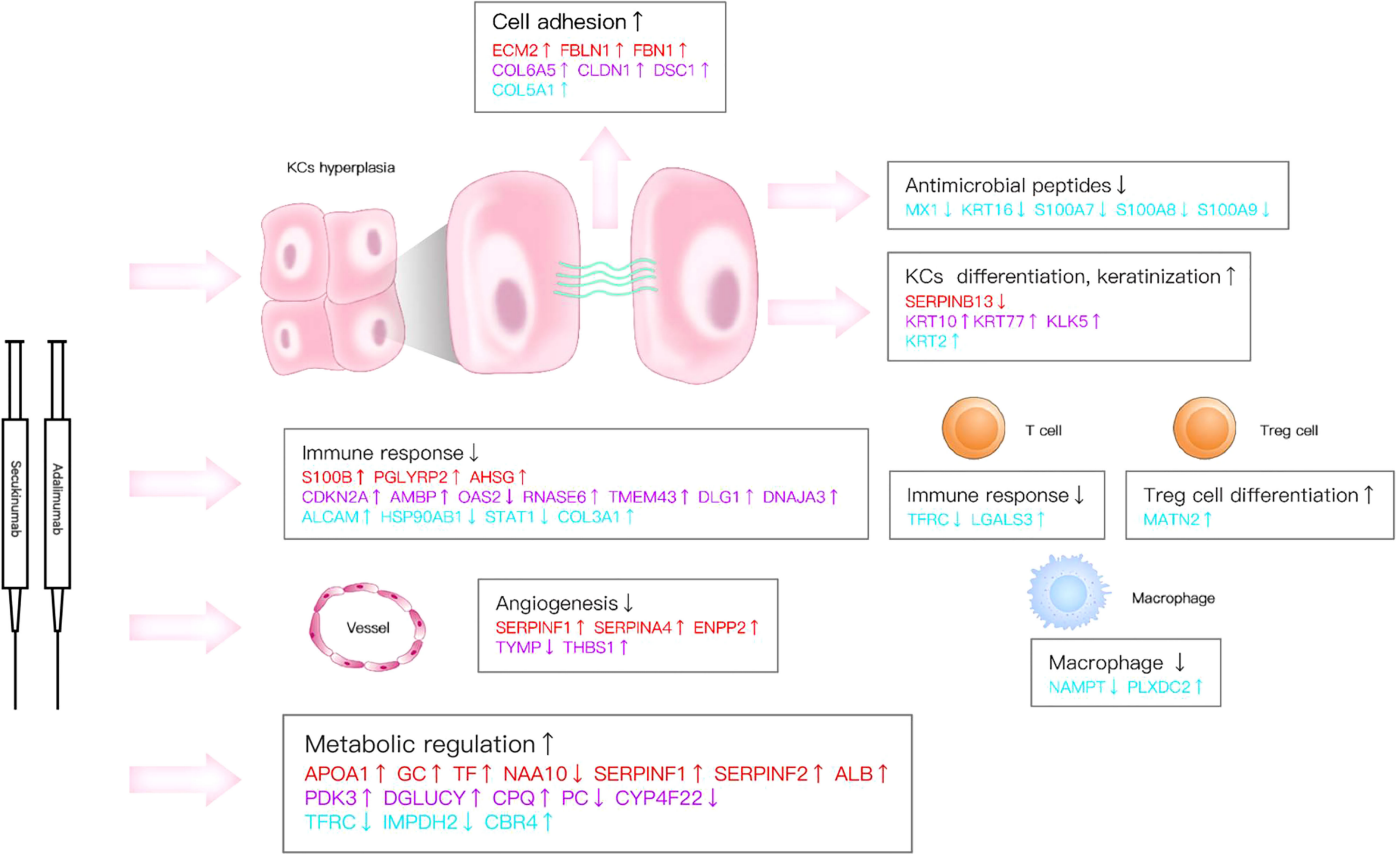
Key DEPs represent multiple molecule changes in psoriasis posttreatment. The figure shows only DEPs with FC>1.5. Proteins are involved in KC function, matrix components, metabolism and immune response based on their corresponding expression levels in the two biological agent groups. Red colour for protein names indicates the adalimumab group. Purple colour indicates the secukinumab group, and blue colour indicates the DEPs in both groups.

Here, we reported a remarkable efficacy of biological agents in psoriasis, which is consistent with previous studies (2, 5). Our research verified that the inhibition of IL-17A and TNF-α can induce multiple molecular changes in psoriatic lesions and have overlapping influences on immune responses and processes, such as different kinds of signalling pathways on immune responses and processes, through direct or indirect effects (**Figures 3B**, **4B**, **5**, **7C**, **8**).

We quantified up to 6054 proteins through the use of DIA-MS, the development of which was a major advance for global protein quantification across multiple samples. In our research, the FC of DEPs was significantly lower than that in a previously reported transcriptomic study (19) because proteins are the final effector molecules in biological systems, while significant differences in their corresponding mRNA levels can exist (20, 21).

Our data revealed new insights into molecular changes in psoriatic skin after the inhibition of IL-17A and TNF-α and identified some important DEPs that participate in immune regulation and biological processes. For example, heat shock protein HSP 90-beta (HSP90AB1) is involved in multiple pathways and processes of cells (22) and is vital for IL-17A-mediated signalling. A previous report found that the HSP90 inhibitor RGRN-305 significantly reduced the IL-17A- and TNF-α- induced proinflammatory genes in human keratinocytes *in vitro* (23). In our study, HSP90 was downregulated after biological treatment and was positively correlated with the PASI score. Signal transducer and activator of transcription 1 (STAT1) was also downregulated; STAT1 mediates cellular responses to interferons (IFNs) and cytokines, acts as a suppressor of NF-κB (24) and synergizes with IL-17. In addition, critical downstream molecules, including S100A7, S100A8, and S100A9, act as antimicrobial peptides and chemotactic factors and play an essential role in innate immunity. Studies have shown that these proteins are associated with disease severity in psoriasis patients (25, 26). In our study, they were all downregulated which indicated a suppression of the immune response and a reduction in disease severity after treatment in both biological agent groups.

Although the drug targets were entirely different, some DEPs were involved in many common immune pathways or biological processes, such as LXR/RXR activation, acute phase response signalling, the MSP-RON signalling pathway, the role of IL-17A in psoriasis, and IL-12 signalling and production in macrophages. We found that eleven proteins (PLXDC2, IMPDH2, ALCAM, HSP90AB1, GAA, COL3A1, PCNA, LGALS3, MATN2, NAMPT, and CBR4) were altered in both groups, and their expression tended to be the same. For instance, galectin 3 (LGALS3) and CD166 (ALCAM) were upregulated in both groups. Research has shown that LGALS3 and CD166 can both bind to CD6. LGALS3 can interfere with the events induced by the CD6-CD166/ALCAM adhesive interactions, followed by T-cell proliferation and cell adhesion (27). Itolizumab, an antibody to CD6, has been applied to treat moderate to severe psoriasis and is effective and well tolerated (28). These proteins were analysed by IPA, and the results showed that they can affect some important pathways, such as the TNF, PI3K-AKT, ERK, NF-κB (complex), IL-17R, MAPK, and interferon-alpha signalling pathways, by direct or indirect action. Therefore, they may be a focus for further study in psoriasis.

Nograles KE et al. found that IL-17 and IL-22 mediate distinct downstream pathways that contribute to psoriatic phenotype (29). Krueger JG et al. used gene set variation analysis (GSVA) to reveal changes in many cell type-specific and inflammatory genes and pathways important in psoriasis (19) after secukinumab therapy. The same results were found in studies by Foulkes AC et al. (13) and Xu M et al. (11) at the serum proteome level after treatment with etanercept and traditional Chinese medicine, respectively. In our research, IPA canonical pathway analysis showed that the separate pathways in the adalimumab group were mainly related to metabolism, such as arginine degradation and oxidative phosphorylation, while in the secukinumab group, the separate pathways primarily focused on the immune system

A previous study showed that an IL17A inhibitor has a neutral impact on metabolic parameters (30), but another study identified changes in genes that regulate peripheral lipid metabolism (19). In our study, metabolic pathways were not activated in the secukinumab group, although some proteins were involved. The reason for the inconsistent results may be the different detection specimens and methods. Therefore, further study is needed to determine whether biological therapy improves the metabolic status and metabolic complications of patients.

Our study also found few DEPs enriched in keratinization that were associated with keratinocyte function and the immune response. Moran et al. found that correcting the dysregulated Th17:Treg axis in HS patients treated with adalimumab (31) provided a rationale for targeting IL-17 in this inflammatory keratosis disease (32). In addition, our data provide clues to phenotypic transformation during biological treatment. For example, kallikrein 5 (KLK5) is overexpressed after secukinumab treatment. KLK5 is a serine protease involved in cell renewal and regulation of skin barrier function (33), and abnormal KLK-5 expression and the hyperactivation of KLK-5 are known to induce atopic dermatitis-like lesions in the skin (34). Some important proteins involved in keratinization, such as KRT6 and KRT17, which have been reported to play a role in psoriasis in previous studies using the IMQ-psoriasis mouse model (35), were not identified in this study. We speculate that this may be due to the different intervention drugs, models and detection methods used.

Studies have found that TNF blockade does not affect or even promote the protein levels of IL-17 (36, 37). Another study found limited effects on alterations in TNF levels in patients treated with secukinumab (38). Krueger et al. found that IL-17A, in synergy with TNF, induces inflammatory products in KCs and ultimately representative psoriatic plaques (19). These findings suggested that the reduced skin inflammation observed in patients after blocking IL-17A is independent of changes in TNF levels and vice versa. Considering that the relevant cytokines and their changes were not detected in this study, we cannot make a direct judgement on this. Nevertheless, we identified an overlapping effect among some proteins on signalling pathways and immune response processes at the proteomic level. Furthermore, it has been shown that bispecific anti-TNF-α/IL-17 antibodies may have superior efficacy and safety in treating rheumatoid arthritis (39), but this has not been demonstrated in another study involving patients with psoriasis arthritis (40). This may be due to partly different pathogenesis between them. Therefore, we suggest that in treating psoriasis, the co-use of these inhibitors may achieve a better effect than the use of a single agent.

This study did not identify two well-known cytokines that play an essential role in the pathogenesis of psoriasis and as drug targets (TNF-α and IL-17A). Similar results were found in another serum proteomic study, in which the authors attributed the lack of the identification of these cytokines to the pathophysiologic heterogeneity of psoriasis disease in the population (40). We think that the lack of detection of these cytokines in DIA and PRM analyses may be due to the limitation of DIA for detecting low-abundance proteins (41). To overcome this limitation, future research would fuse targeted proteomics (such as MS3-based methods) or commercial protein microarrays with DIA to reliably identify high- and low-abundance proteins.

## Limitations of the study

(1) The sample size was relatively small. (2) Some vital low-abundance proteins were not detected because of limited detection ability by DIA. (3) The median age and course of the disease were lower in the adalimumab group (**Table 1**), so their impact on our data interpretation could not be precisely defined. To a certain extent, these confounding factors might influence the research results.

## Conclusions

In conclusion, this study presents a systematic proteomic investigation of skin samples from two biological agent groups with different mechanisms of action and a control group of psoriasis patients. The results indicated that inhibiting these molecules could induce multiple molecular changes in psoriatic lesions and have overlapping influences on immune responses and processes, such as different kinds of signalling pathways and immune processes, through direct or indirect effects. Our results suggested that the combined use of these two inhibitors may provide better efficacy in treating psoriasis.

## Data availability statement

The data presented in the study are deposited in the iProX repository (http://proteomecentral.proteomexchange.org/cgi/GetDataset?ID=PXD037579), accession number PXD037579.

## Ethics statement

The studies involving human participants were reviewed and approved by Dermatology Hospital of Zhejiang Province (LL-2020-15). The patients/participants provided their written informed consent to participate in this study.

## Author contributions

QD and DL designed and supervised the project. QD wrote the manuscript with input from the authors. BBX, XXJ, YLT, GHL, NNS, and XBY. Collected the samples and clinical data. LHH and JH preserved the samples. All authors contributed to the article and approved the submitted version.

## Acknowledgments

The authors thank Genechem Co., Ltd. (Shanghai, China) for the mass spectrometric analysis. We thank Dr Sun. for data computation.

## Conflict of interest

The authors declare that the research was conducted in the absence of any commercial or financial relationships that could be construed as a potential conflict of interest.

## Publisher’s note

All claims expressed in this article are solely those of the authors and do not necessarily represent those of their affiliated organizations, or those of the publisher, the editors and the reviewers. Any product that may be evaluated in this article, or claim that may be made by its manufacturer, is not guaranteed or endorsed by the publisher.

## Supplementary material

Supplementary Figure 1 Quality control: Normalization of DIA quantification results. Before normalization **(A)**. After normalization **(B)**.

Supplementary Figure 2 Quality control: CV distribution diagram of protein.

Supplementary Figure 3 The confounding DEGs at baseline were excluded using a Venn diagram analysis. Venn diagrams show the overlaps between special proteins (proteins are specifically differentially expressed at one point in time in each group without at baseline) and proteins with time trends. Overlapping proteins will be screened for further study.

Supplementary Figure 4 PASI score curve for clinical improvement of patients in adalimumab (ADM), secukinumab (BJS), controls (AWJ), N=9 per group. The ordinate represents the PASI score, and the abscissa represents different treatment time points. Curves in different colours represent different groups. Paired two-tailed Student’s t tests were used to assess statistical significance. *p < 0.05, **p < 0.01, ***p < 0.001, ****p < 0.0001.

Supplementary Figure 5 The significantly altered pathways in the adalimumab **(A)** and secukinumab groups **(B)**. Ingenuine pathway analysis (IPA) was performed for pathway analysis. The colour represents the Z score by IPA and the predicted activation or suppression state.

## Abbreviations

TNF, tumour necrosis factor; PASI, Psoriasis Area and Severity Index; LS, lesional; CV, coefficient variance; STEM, Short Time-series Expression Miner; STAT1, signal transducer and activator of transcription; ARG1, Arginase 1; MSP, Macrophage stimulating protein; RON, Recepteur d’origine nantais; LXR, liver X receptor; FXR, farnesoid X receptor; IFNs, interferons; HS, Hidradenitis Suppurativa; KLK5, Kallikrein 5; IBD, inflammatory bowel disease; MYL9, Myosin regulatory light polypeptide 9.
