# Supplementary figures and images for: IL-17A and TNF-α inhibitors induce multiple molecular changes in psoriasis
Source: Front Immunol. 2022 Nov 22;13:1015182. doi: 10.3389/fimmu.2022.1015182 (PMC9723344; doi:10.3389/fimmu.2022.1015182)

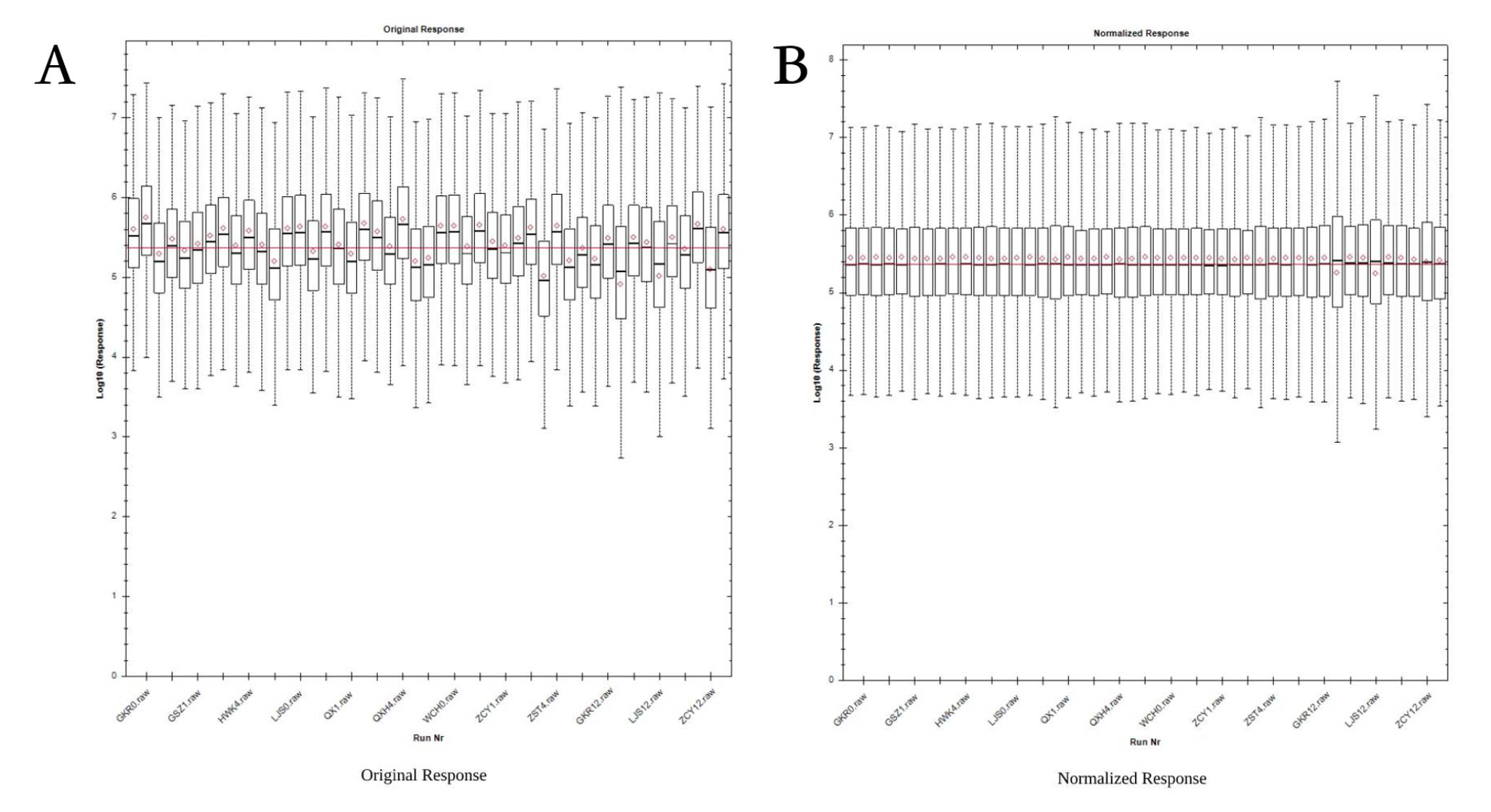

Supplement: Supplementary Figure 1 — Quality control: Normalization of DIA quantification results. Before normalization (A). After normalization (B). [file Image_1.jpg]

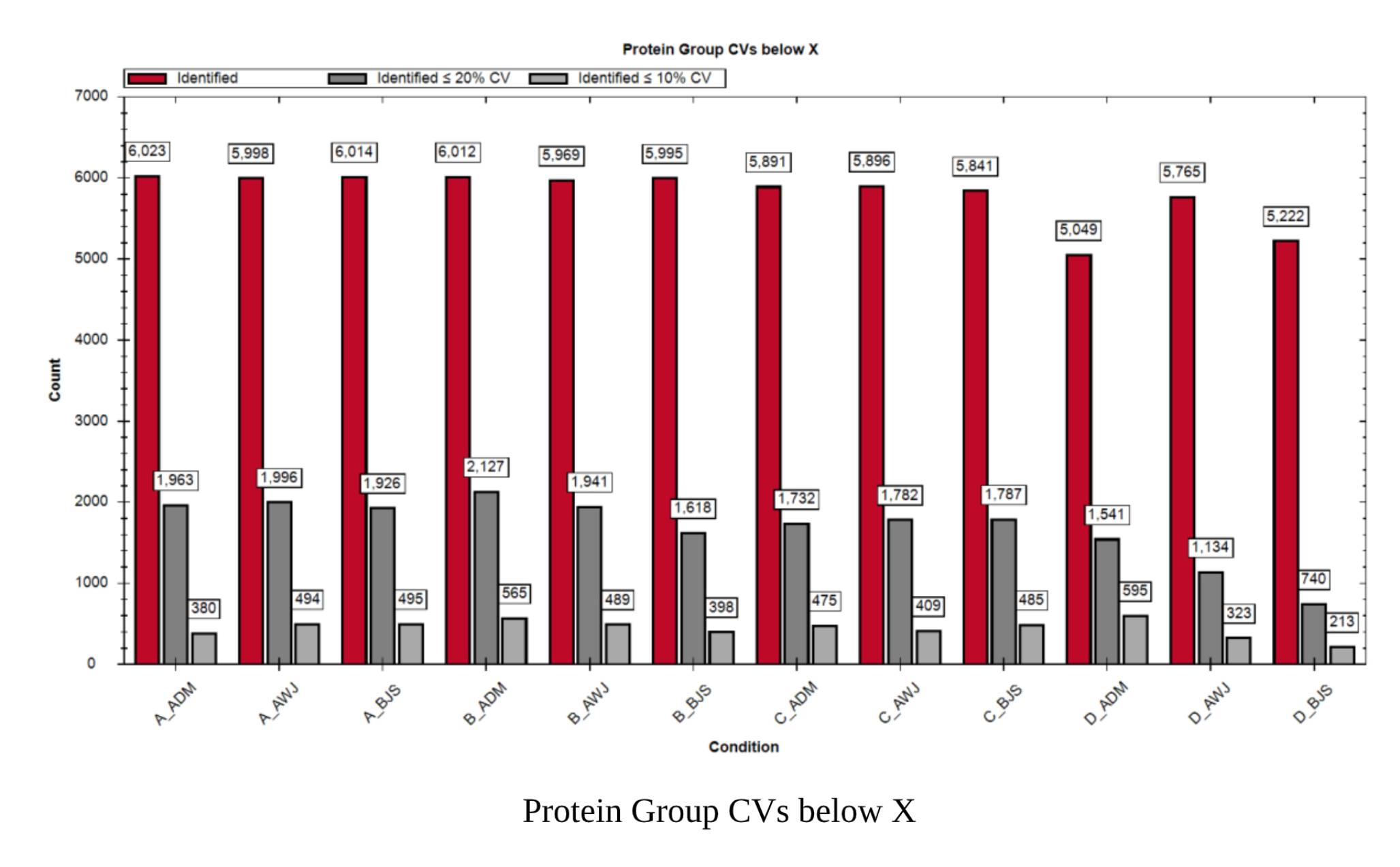

Supplement: Supplementary Figure 2 — Quality control: CV distribution diagram of protein. [file Image_2.jpg]

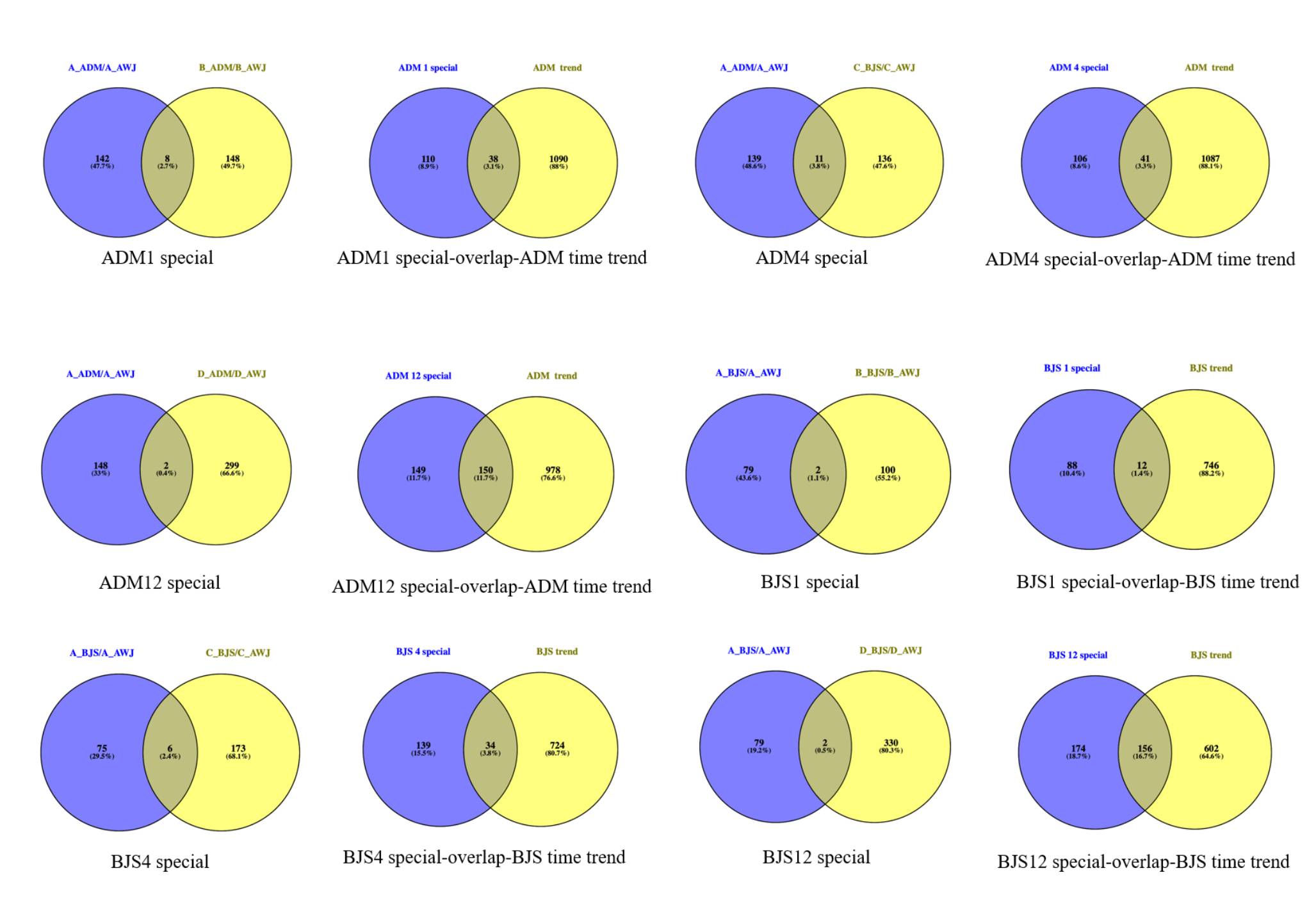

Supplement: Supplementary Figure 3 — The confounding DEGs at baseline were excluded using a Venn diagram analysis. Venn diagrams show the overlaps between special proteins (proteins are specifically differentially expressed at one point in time in each group without at baseline) and proteins with time trends. Overlapping proteins will be screened for further study. [file Image_3.jpg]

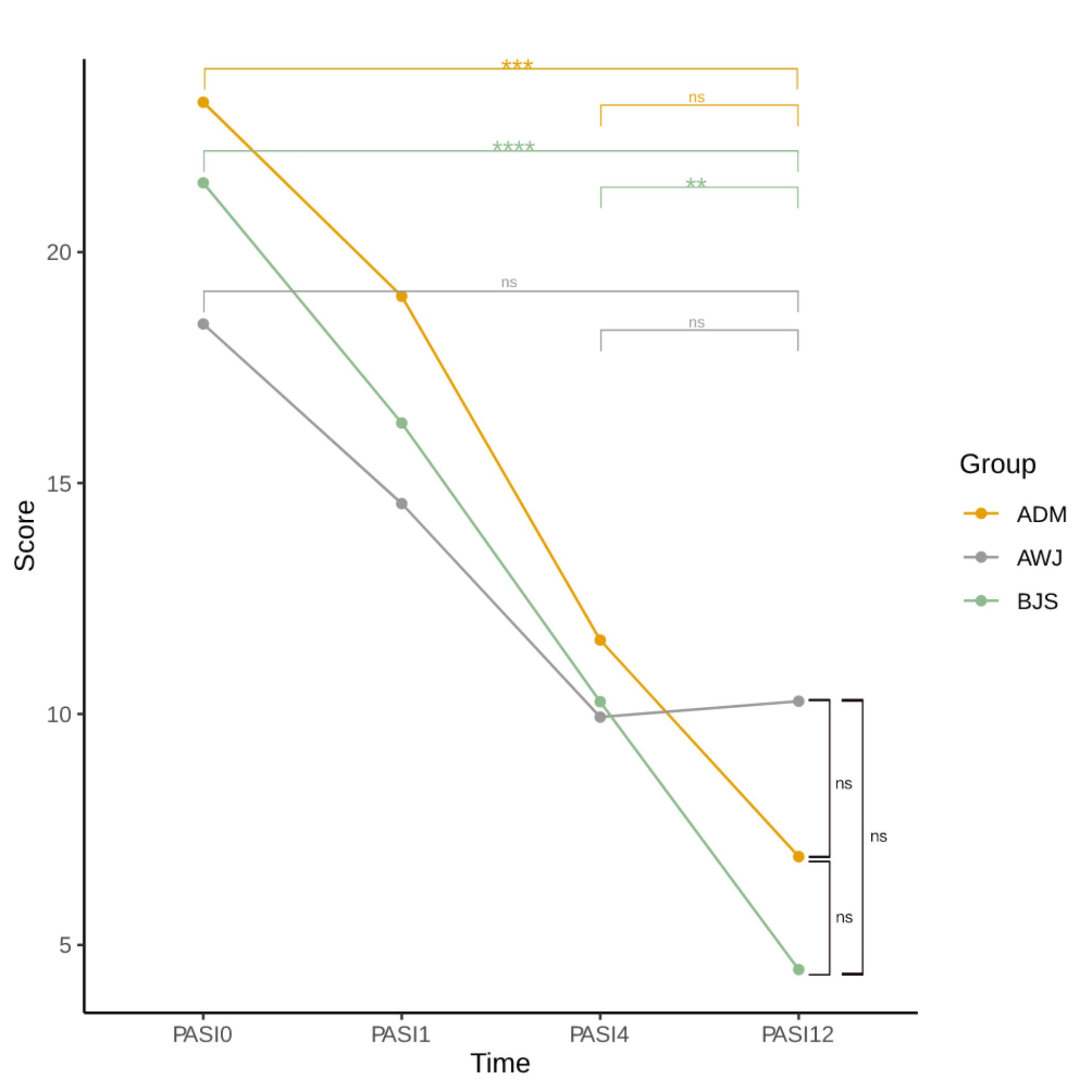

Supplement: Supplementary Figure 4 — PASI score curve for clinical improvement of patients in adalimumab (ADM), secukinumab (BJS), controls (AWJ), N=9 per group. The ordinate represents the PASI score, and the abscissa represents different treatment time points. Curves in different colours represent different groups. Paired two-tailed Student’s t tests were used to assess statistical significance. *p < 0.05, **p < 0.01, ***p < 0.001, ****p < 0.0001. [file Image_4.jpg]

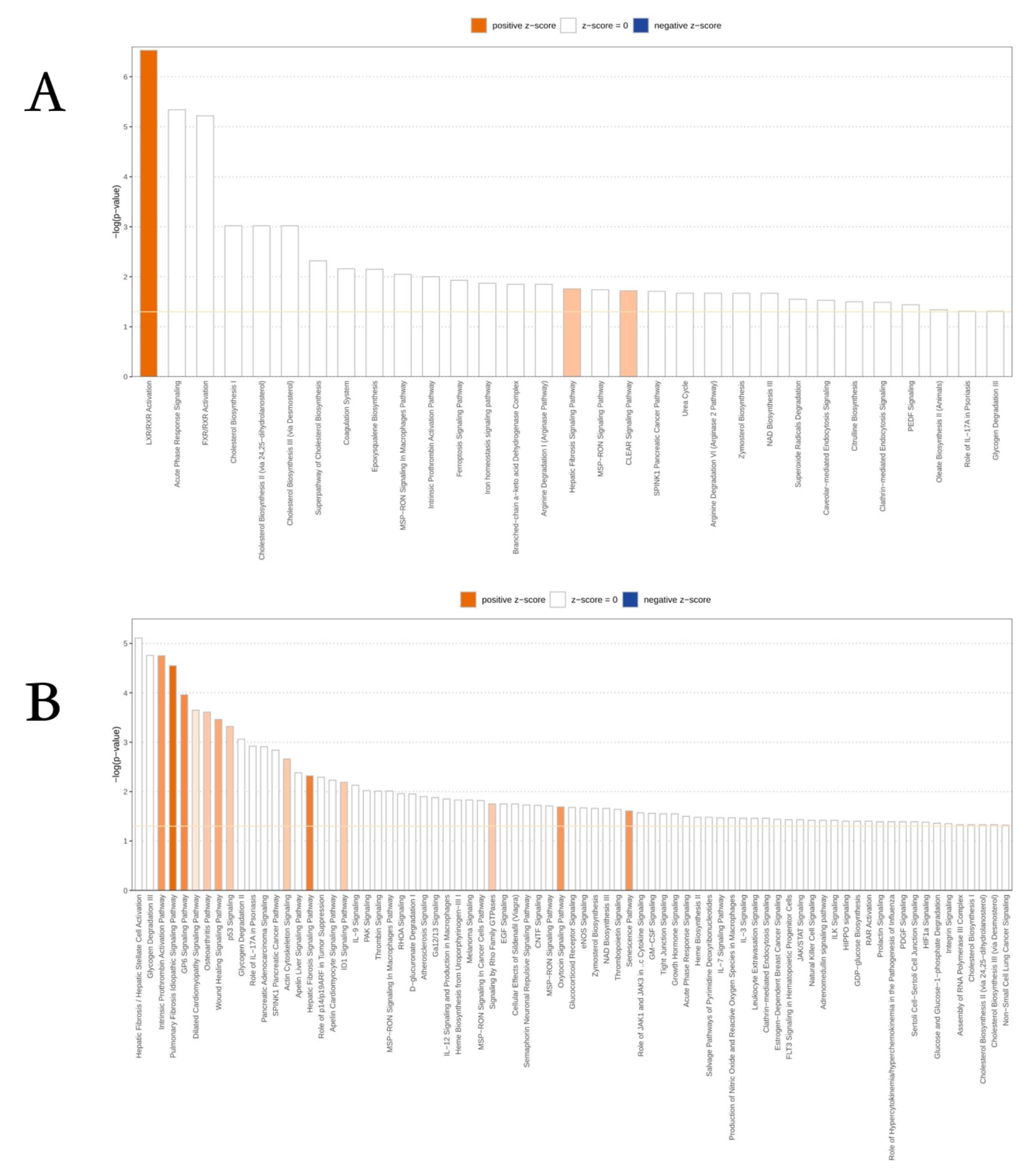

Supplement: Supplementary Figure 5 — The significantly altered pathways in the adalimumab (A) and secukinumab groups (B). Ingenuine pathway analysis (IPA) was performed for pathway analysis. The colour represents the Z score by IPA and the predicted activation or suppression state. [file Image_5.jpg]
