# Supplementary material for: IL-17A and TNF-α inhibitors induce multiple molecular changes in psoriasis
Source: Front Immunol. 2022 Nov 22;13:1015182. doi: 10.3389/fimmu.2022.1015182 (PMC9723344; doi:10.3389/fimmu.2022.1015182)
Supplement: Supplementary file 6 [file Table_1.docx]

**Table S1** Early and Lately altered protein in each group

Early up-regulation in ADM

| **PG.ProteinAccessions** | **GENE Names** | **SPOT** | **Profile** | **A_ADM** | **B_ADM** | **C_ADM** | **D_ADM** |
| --- | --- | --- | --- | --- | --- | --- | --- |
| Q9H2D6 | TRIOBP | ID_1541 | 16 | 0 | -0.05 | 2.1 | 6.27 |
| O15067 | PFAS | ID_74 | 16 | 0 | 0.97 | 2.3 | 6 |
| P43115 | PTGER3 | ID_696 | 25 | 0 | 1.53 | 4.03 | 5.18 |
| Q9H2Y9 | SLCO5A1 | ID_1545 | 16 | 0 | -0.49 | 2.6 | 4.97 |
| Q15286 | RAB35 | ID_1073 | 16 | 0 | 0.02 | 3.13 | 4.56 |
| Q13393 | PLD1 | ID_984 | 16 | 0 | 0.31 | 2.86 | 4.33 |
| Q8IUR6 | CREBRF | ID_1256 | 16 | 0 | 0.4 | 1.61 | 4.16 |
| A0A075B6S2 | IGKV2D-29;IGKV2-29 | ID_1 | 16 | 0 | -0.08 | 1.14 | 4.02 |
| Q14207 | NPAT | ID_1027 | 25 | 0 | 1.12 | 2.56 | 3.95 |
| P50135 | HNMT | ID_743 | 16 | 0 | 0.03 | 1.31 | 3.93 |
| Q13884 | SNTB1 | ID_1011 | 16 | 0 | 0.15 | 1.49 | 3.86 |
| Q15057 | ACAP2 | ID_1061 | 16 | 0 | 0.46 | 2.13 | 3.75 |
| P21817 | RYR1 | ID_532 | 16 | 0 | 0.23 | 1.67 | 3.7 |
| Q96BJ3 | AIDA | ID_1388 | 16 | 0 | 0.14 | 1.12 | 3.7 |
| Q5D862 | FLG2 | ID_1142 | 25 | 0 | 0.74 | 2.64 | 3.56 |
| A0A1B0GTW7 | LMLN2 | ID_17 | 25 | 0 | 1.3 | 2.15 | 3.46 |
| Q6NUJ1 | PSAPL1 | ID_1173 | 16 | 0 | -0.11 | 2.03 | 3.41 |
| Q58EX7 | PLEKHG4 | ID_1139 | 25 | 0 | 1.21 | 2.42 | 3.31 |
| P09105 | HBQ1 | ID_386 | 25 | 0 | 0.75 | 1.63 | 3.22 |
| P29017 | CD1C | ID_588 | 25 | 0 | 1.11 | 2.38 | 3.16 |
| Q8IW75 | SERPINA12 | ID_1260 | 16 | 0 | 0.32 | 1.93 | 3.1 |
| Q8IZU8 | DSEL | ID_1281 | 16 | 0 | -0.15 | 0.98 | 3.1 |
| Q9BRX8 | PRXL2A | ID_1470 | 16 | 0 | 0.22 | 1.07 | 3.1 |
| Q7Z3B1 | NEGR1 | ID_1224 | 25 | 0 | 0.56 | 1.98 | 3.09 |
| P13716 | ALAD | ID_452 | 16 | 0 | 0.21 | 1.09 | 3.04 |
| Q15417 | CNN3 | ID_1081 | 16 | 0 | 0.13 | 0.85 | 3.01 |
| P02042 | HBD | ID_269 | 25 | 0 | 0.67 | 1.62 | 2.97 |
| P04040 | CAT | ID_297 | 16 | 0 | 0.5 | 1.24 | 2.84 |
| Q08345 | DDR1 | ID_945 | 16 | 0 | 0.2 | 1.31 | 2.84 |
| P16157 | ANK1 | ID_482 | 25 | 0 | 1.12 | 1.65 | 2.83 |
| P05452 | CLEC3B | ID_322 | 16 | 0 | -0.18 | 1.44 | 2.82 |
| P01019 | AGT | ID_250 | 16 | 0 | 0.02 | 1.05 | 2.81 |
| Q7Z7G8 | VPS13B | ID_1238 | 16 | 0 | -0.19 | 1.18 | 2.81 |
| Q99766 | DMAC2L | ID_1458 | 16 | 0 | 0.51 | 1.42 | 2.77 |
| P02774 | GC | ID_288 | 16 | 0 | 0.13 | 1.24 | 2.76 |
| P02647 | APOA1 | ID_277 | 16 | 0 | 0.12 | 1.18 | 2.75 |
| P15090 | FABP4 | ID_468 | 16 | 0 | 0.17 | 0.95 | 2.75 |
| Q9BX66 | SORBS1 | ID_1498 | 16 | 0 | 0.29 | 1.02 | 2.75 |
| Q93100 | PHKB | ID_1374 | 25 | 0 | 0.98 | 2.08 | 2.74 |
| P55064 | AQP5 | ID_792 | 16 | 0 | 0.02 | 1.08 | 2.73 |
| Q9P283 | SEMA5B | ID_1662 | 16 | 0 | 0.39 | 1.84 | 2.73 |
| P69905 | HBA2 | ID_873 | 25 | 0 | 0.8 | 1.79 | 2.72 |
| Q08431 | MFGE8 | ID_947 | 16 | 0 | 0.26 | 1.53 | 2.71 |
| Q15121 | PEA15 | ID_1065 | 16 | 0 | 0.54 | 1.1 | 2.71 |
| Q8N6Y2 | LRRC17 | ID_1299 | 16 | 0 | 0.42 | 0.88 | 2.7 |
| A0A0C4DH25 | IGKV3D-20 | ID_7 | 16 | 0 | 0.46 | 1.17 | 2.69 |
| Q13332 | PTPRS | ID_981 | 16 | 0 | 0.06 | 1.32 | 2.69 |
| Q9UGT4 | SUSD2 | ID_1692 | 16 | 0 | 0.05 | 1.22 | 2.68 |
| P35443 | THBS4 | ID_644 | 16 | 0 | -0.47 | 0.58 | 2.67 |
| P00918 | CA2 | ID_248 | 25 | 0 | 0.6 | 1.42 | 2.66 |
| P25311 | AZGP1 | ID_561 | 16 | 0 | 0.14 | 1.5 | 2.66 |
| Q6JBY9 | RCSD1 | ID_1171 | 25 | 0 | 0.83 | 1.67 | 2.66 |
| P13671 | C6 | ID_450 | 16 | 0 | 0.09 | 0.82 | 2.65 |
| O14495 | PLPP3 | ID_53 | 16 | 0 | 0.54 | 0.95 | 2.64 |
| Q13769 | THOC5 | ID_1005 | 16 | 0 | -0.09 | 1.06 | 2.64 |
| P29622 | SERPINA4 | ID_597 | 16 | 0 | 0.16 | 0.78 | 2.63 |
| P01593 | IGKV1D-33;IGKV1-33 | ID_257 | 25 | 0 | 0.76 | 1.3 | 2.62 |
| P02775 | PPBP | ID_289 | 25 | 0 | 0.96 | 1.97 | 2.59 |
| P02462 | COL4A1 | ID_273 | 16 | 0 | 0.42 | 0.84 | 2.57 |
| Q9H9S4 | CAB39L | ID_1573 | 25 | 0 | 0.68 | 2.19 | 2.56 |
| B9A064 | IGLL5 | ID_27 | 16 | 0 | -0.07 | 0.93 | 2.55 |
| P27169 | PON1 | ID_577 | 16 | 0 | 0.5 | 1.22 | 2.55 |
| P30711 | GSTT1 | ID_615 | 16 | 0 | 0.02 | 0.71 | 2.54 |
| P68871 | HBB | ID_871 | 25 | 0 | 0.59 | 1.61 | 2.53 |
| Q96P44 | COL21A1 | ID_1427 | 16 | 0 | 0.13 | 1.12 | 2.53 |
| Q7Z794 | KRT77 | ID_1237 | 16 | 0 | 0.45 | 1.4 | 2.51 |
| P02461 | COL3A1 | ID_272 | 16 | 0 | 0.41 | 1.48 | 2.5 |
| Q86UA1 | PRPF39 | ID_1246 | 16 | 0 | 0.33 | 0.79 | 2.49 |
| Q9UGM5 | FETUB | ID_1690 | 16 | 0 | 0.07 | 0.76 | 2.49 |
| Q9Y646 | CPQ | ID_1785 | 25 | 0 | 0.51 | 1.33 | 2.47 |
| P02760 | AMBP | ID_284 | 25 | 0 | 0.48 | 1.38 | 2.45 |
| P02753 | RBP4 | ID_283 | 16 | 0 | 0.24 | 0.98 | 2.44 |
| Q14CM0 | FRMPD4 | ID_1050 | 16 | 0 | 0.27 | 0.99 | 2.42 |
| P01008 | SERPINC1 | ID_249 | 16 | 0 | 0.13 | 1.11 | 2.41 |
| P01624 | IGKV3-15 | ID_259 | 16 | 0 | 0.49 | 0.95 | 2.41 |
| P02654 | APOC1 | ID_280 | 16 | 0 | 0.36 | 0.81 | 2.41 |
| P02768 | ALB | ID_287 | 16 | 0 | 0.09 | 1.53 | 2.4 |
| P01042 | KNG1 | ID_254 | 16 | 0 | 0.24 | 0.86 | 2.39 |
| Q05193 | DNM1 | ID_929 | 16 | 0 | 0.22 | 1.14 | 2.39 |
| Q6R327 | RICTOR | ID_1188 | 16 | 0 | -0.03 | 1.47 | 2.39 |
| Q8N2G8 | GHDC | ID_1287 | 16 | 0 | 0.12 | 0.96 | 2.39 |
| P12273 | PIP | ID_439 | 16 | 0 | 0.43 | 1.1 | 2.38 |
| Q6WCQ1 | MPRIP | ID_1198 | 16 | 0 | 0 | 0.75 | 2.38 |
| P26927 | MST1 | ID_575 | 16 | 0 | 0.16 | 1.16 | 2.37 |
| P02765 | AHSG | ID_286 | 16 | 0 | -0.01 | 0.92 | 2.36 |
| Q13228 | SELENBP1 | ID_973 | 16 | 0 | 0.16 | 0.93 | 2.35 |
| P24592 | IGFBP6 | ID_557 | 16 | 0 | 0.01 | 0.75 | 2.34 |
| P32119 | PRDX2 | ID_626 | 16 | 0 | 0.36 | 0.94 | 2.32 |
| Q9BZG8 | DPH1 | ID_1518 | 16 | 0 | -0.44 | 1.28 | 2.31 |
| P51888 | PRELP | ID_759 | 16 | 0 | 0.29 | 1.19 | 2.29 |
| P69891 | HBG1 | ID_872 | 16 | 0 | 0.1 | 1.6 | 2.29 |
| Q14624 | ITIH4 | ID_1035 | 25 | 0 | 0.65 | 1.26 | 2.28 |
| Q15828 | CST6 | ID_1095 | 16 | 0 | 0.02 | 1.35 | 2.28 |
| P07358 | C8B | ID_347 | 16 | 0 | 0.05 | 1.03 | 2.27 |
| P07951 | TPM2 | ID_357 | 16 | 0 | 0.48 | 0.81 | 2.25 |
| Q13464 | ROCK1 | ID_989 | 16 | 0 | 0.14 | 0.7 | 2.22 |
| P30837 | ALDH1B1 | ID_617 | 16 | 0 | 0.34 | 0.75 | 2.19 |
| A0A075B7D8 | IGHV3OR15-7 | ID_2 | 16 | 0 | -0.23 | 0.8 | 2.18 |
| P05155 | SERPING1 | ID_316 | 16 | 0 | -0.15 | 0.62 | 2.18 |
| Q13515 | BFSP2 | ID_993 | 16 | 0 | 0.02 | 0.87 | 2.18 |
| Q14956 | GPNMB | ID_1046 | 16 | 0 | -0.26 | 0.59 | 2.18 |
| P78537 | BLOC1S1 | ID_879 | 16 | 0 | 0.28 | 1.35 | 2.17 |
| Q13795 | ARFRP1 | ID_1006 | 16 | 0 | -0.03 | 1.08 | 2.17 |
| Q16610 | ECM1 | ID_1107 | 16 | 0 | -0.2 | 1.28 | 2.17 |
| Q9BVQ7 | SPATA5L1 | ID_1495 | 16 | 0 | -0.08 | 0.7 | 2.17 |
| P08514 | ITGA2B | ID_368 | 25 | 0 | 0.76 | 1.27 | 2.15 |
| P14672 | SLC2A4 | ID_463 | 16 | 0 | 0.19 | 0.92 | 2.15 |
| Q99808 | SLC29A1 | ID_1460 | 16 | 0 | 0.31 | 0.77 | 2.15 |
| P07738 | BPGM | ID_352 | 16 | 0 | 0.25 | 0.94 | 2.14 |
| Q5JSZ5 | PRRC2B | ID_1143 | 16 | 0 | -0.68 | 1.43 | 2.12 |
| P01700 | IGLV1-47 | ID_260 | 16 | 0 | -0.02 | 0.77 | 2.11 |
| P78559 | MAP1A | ID_880 | 16 | 0 | 0.34 | 0.68 | 2.11 |
| Q5SYB0 | FRMPD1 | ID_1153 | 16 | 0 | -0.43 | 1.19 | 2.11 |
| Q9UEW8 | STK39 | ID_1685 | 16 | 0 | -0.28 | 1.02 | 2.1 |
| Q14CZ7 | FASTKD3 | ID_1051 | 16 | 0 | 0.16 | 1.38 | 2.09 |
| Q4UJ75 | ANKRD20A4;ANKRD20A8P;ANKRD20A2;ANKRD20A1;ANKRD20A3 | ID_1132 | 16 | 0 | 0.4 | 1.08 | 2.09 |
| O75106 | AOC2 | ID_144 | 16 | 0 | 0.32 | 1.34 | 2.08 |
| P23297 | S100A1 | ID_549 | 25 | 0 | 0.56 | 1.42 | 2.08 |
| P02787 | TF | ID_291 | 16 | 0 | -0.04 | 0.88 | 2.06 |
| P01782 | IGHV3-9;IGHV3-43D | ID_263 | 16 | 0 | 0.15 | 0.69 | 2.05 |
| P08697 | SERPINF2 | ID_375 | 16 | 0 | 0.21 | 0.85 | 2.03 |
| P0DP01 | IGHV1-8 | ID_404 | 16 | 0 | -0.33 | 0.44 | 2.03 |
| Q15113 | PCOLCE | ID_1064 | 16 | 0 | 0.08 | 1.37 | 2.03 |
| Q9HBI1 | PARVB | ID_1581 | 16 | 0 | 0.29 | 1.27 | 2.03 |
| A0A0A0MRZ8 | IGKV3D-11;IGKV3-11 | ID_3 | 16 | 0 | 0.17 | 0.92 | 2.02 |
| P49247 | RPIA | ID_728 | 16 | 0 | 0.39 | 0.83 | 2.02 |
| Q9P2X3 | IMPACT | ID_1670 | 16 | 0 | 0.12 | 0.69 | 2.02 |
| Q9Y680 | FKBP7 | ID_1789 | 16 | 0 | 0.39 | 0.94 | 2.01 |
| Q9Y2J8 | PADI2 | ID_1746 | 16 | 0 | -0.32 | 0.82 | 2 |

Lately up-regulation in ADM

| **PG.ProteinAccessions** | **GENE Names** | **SPOT** | **Profile** | **A_ADM** | **B_ADM** | **C_ADM** | **D_ADM** |
| --- | --- | --- | --- | --- | --- | --- | --- |
| P29317 | EPHA2 | ID_591 | 13 | 0 | -0.04 | 2.25 | 9.23 |
| P05549 | TFAP2A | ID_326 | 13 | 0 | 0.83 | -0.14 | 6.93 |
| Q5VST9 | OBSCN | ID_1158 | 13 | 0 | 1.01 | -1.19 | 6.43 |
| Q6ZNJ1 | NBEAL2 | ID_1203 | 13 | 0 | -0.05 | -0.14 | 5.85 |
| Q96EP0 | RNF31 | ID_1402 | 13 | 0 | 1.34 | 1.58 | 5.72 |
| Q9H2K8 | TAOK3 | ID_1543 | 13 | 0 | 0.01 | 0.65 | 5.49 |
| Q8IWB7 | WDFY1 | ID_1262 | 13 | 0 | -0.16 | 0.08 | 4.98 |
| P30419 | NMT1 | ID_608 | 13 | 0 | 0.24 | 0.15 | 4.68 |
| Q14699 | RFTN1 | ID_1040 | 13 | 0 | 0.19 | 0.28 | 4.62 |
| P42858 | HTT | ID_694 | 13 | 0 | 0.41 | -0.04 | 4.54 |
| O60508 | CDC40 | ID_124 | 13 | 0 | -0.24 | -0.62 | 4.43 |
| P29590 | PML | ID_596 | 13 | 0 | -0.05 | 0.03 | 4.39 |
| Q6ZS81 | WDFY4 | ID_1205 | 13 | 0 | -1.16 | -1.87 | 3.81 |
| P42773 | CDKN2C | ID_693 | 13 | 0 | 0.82 | 0.91 | 3.69 |
| Q15386 | UBE3C | ID_1079 | 13 | 0 | 0.01 | 0.08 | 3.67 |
| O75094 | SLIT3 | ID_143 | 13 | 0 | -0.23 | -0.25 | 3.66 |
| Q5SRH9 | TTC39A | ID_1152 | 13 | 0 | 0.35 | -0.72 | 3.64 |
| P43652 | AFM | ID_702 | 13 | 0 | -0.05 | 0.7 | 3.58 |
| Q96HW7 | INTS4 | ID_1414 | 13 | 0 | 0.16 | -0.14 | 3.53 |
| O60840 | CACNA1F | ID_137 | 13 | 0 | 0.68 | 1.05 | 3.47 |
| Q16740 | CLPP | ID_1112 | 13 | 0 | 0.17 | -0.41 | 3.44 |
| P07477 | PRSS1 | ID_349 | 13 | 0 | 0.64 | 0.54 | 3.31 |
| Q13326 | SGCG | ID_980 | 13 | 0 | 0.22 | 0.56 | 3.27 |
| Q8NBJ5 | COLGALT1 | ID_1303 | 13 | 0 | 0.13 | 0.38 | 3.26 |
| A1A4S6 | ARHGAP10 | ID_18 | 13 | 0 | -0.87 | -0.49 | 3.19 |
| Q9C0D3 | ZYG11B | ID_1522 | 13 | 0 | -0.01 | -0.64 | 3.17 |
| Q9NYQ8 | FAT2 | ID_1638 | 13 | 0 | 0.31 | 0.78 | 3.12 |
| A6NMZ7 | COL6A6 | ID_23 | 13 | 0 | 0.32 | 0.88 | 3.07 |
| Q92917 | GPKOW | ID_1367 | 13 | 0 | -1.29 | -0.76 | 3.04 |
| O95969 | SCGB1D2 | ID_228 | 13 | 0 | 0.34 | 0.74 | 2.99 |
| Q4G0N8 | SLC9C1 | ID_1130 | 13 | 0 | -0.56 | -0.14 | 2.88 |
| Q6IN85 | PPP4R3A | ID_1169 | 13 | 0 | -0.22 | -0.27 | 2.87 |
| Q9Y6K1 | DNMT3A | ID_1797 | 13 | 0 | -0.13 | 0.25 | 2.87 |
| O95445 | APOM | ID_210 | 13 | 0 | 0.09 | 0.23 | 2.84 |
| Q9UKE5 | TNIK | ID_1712 | 13 | 0 | -0.13 | -0.37 | 2.84 |
| P0C2W1 | FBXO45 | ID_399 | 13 | 0 | 0.46 | -0.5 | 2.83 |
| Q9BZA7 | PCDH11X | ID_1516 | 13 | 0 | 0.5 | 0.27 | 2.82 |
| Q8IWT0 | ZBTB8OS | ID_1263 | 13 | 0 | 0.23 | -0.43 | 2.8 |
| Q8N126 | CADM3 | ID_1283 | 13 | 0 | 0.02 | 0.71 | 2.79 |
| P36404 | ARL2 | ID_653 | 13 | 0 | 0.02 | 0.29 | 2.77 |
| O00479 | HMGN4 | ID_44 | 13 | 0 | -0.29 | -0.05 | 2.73 |
| Q06732 | ZNF33B | ID_938 | 13 | 0 | 0.3 | 0.8 | 2.69 |
| P31997 | CEACAM8 | ID_625 | 13 | 0 | 0.7 | -0.97 | 2.62 |
| P04271 | S100B | ID_305 | 13 | 0 | 0.43 | 0.51 | 2.59 |
| Q15036 | SNX17 | ID_1056 | 13 | 0 | -0.49 | 0.08 | 2.57 |
| P03952 | KLKB1 | ID_296 | 13 | 0 | 0.33 | 0.57 | 2.56 |
| P37235 | HPCAL1 | ID_662 | 13 | 0 | 0.26 | 0.6 | 2.55 |
| O14558 | HSPB6 | ID_55 | 13 | 0 | 0.06 | 0.6 | 2.49 |
| P02763 | ORM1 | ID_285 | 13 | 0 | -0.19 | 0.41 | 2.46 |
| Q7LBC6 | KDM3B | ID_1220 | 13 | 0 | -0.17 | 0.36 | 2.44 |
| Q8N0X4 | CLYBL | ID_1282 | 13 | 0 | -1.42 | -1.16 | 2.44 |
| Q92820 | GGH | ID_1362 | 13 | 0 | 0.26 | 0.59 | 2.43 |
| Q92522 | H1-10 | ID_1349 | 13 | 0 | -0.03 | 0.45 | 2.42 |
| Q13308 | PTK7 | ID_976 | 13 | 0 | -0.23 | -0.06 | 2.4 |
| P32970 | CD70 | ID_629 | 13 | 0 | 0.31 | 0.39 | 2.37 |
| P30041 | PRDX6 | ID_602 | 13 | 0 | 0.1 | 0.18 | 2.34 |
| Q9Y5Y7 | LYVE1 | ID_1783 | 13 | 0 | -0.5 | 0.05 | 2.34 |
| P00325 | ADH1B | ID_234 | 13 | 0 | 0.05 | 0.5 | 2.32 |
| P22033 | MMUT | ID_535 | 13 | 0 | -0.09 | 0.42 | 2.32 |
| P01619 | IGKV3-20 | ID_258 | 13 | 0 | -0.07 | 0.23 | 2.31 |
| Q9HC73 | CRLF2 | ID_1585 | 13 | 0 | -0.14 | 0.22 | 2.27 |
| Q9BXX0 | EMILIN2 | ID_1510 | 13 | 0 | 0.31 | -0.26 | 2.26 |
| P61204 | ARF3;ARF1 | ID_817 | 13 | 0 | 0.28 | 0.28 | 2.25 |
| Q8WWX9 | SELENOM | ID_1336 | 13 | 0 | 0.2 | 0.29 | 2.25 |
| Q9NR45 | NANS | ID_1608 | 13 | 0 | 0.43 | 0.7 | 2.24 |
| Q8NBM8 | PCYOX1L | ID_1305 | 13 | 0 | 0.56 | 0.35 | 2.23 |
| Q9BTC8 | MTA3 | ID_1480 | 13 | 0 | -0.05 | -0.87 | 2.22 |
| P62314 | SNRPD1 | ID_841 | 13 | 0 | 0.06 | 0.34 | 2.18 |
| Q14118 | DAG1 | ID_1018 | 13 | 0 | -0.16 | 0.18 | 2.17 |
| P08727 | KRT19 | ID_377 | 13 | 0 | 0.05 | 0.43 | 2.1 |
| Q9P0K7 | RAI14 | ID_1655 | 13 | 0 | 0.69 | 0.11 | 2.1 |
| Q9UMS6 | SYNPO2 | ID_1729 | 13 | 0 | 0.43 | 0.4 | 2.09 |
| P00352 | ALDH1A1 | ID_235 | 13 | 0 | 0.01 | 0.31 | 2.05 |
| P02511 | CRYAB | ID_274 | 13 | 0 | 0.35 | 0.63 | 2.05 |
| O60825 | PFKFB2 | ID_135 | 13 | 0 | 0.07 | -0.56 | 2.04 |
| Q9NUJ1 | ABHD10 | ID_1622 | 13 | 0 | 0.27 | 0.26 | 2.03 |
| Q9UK22 | FBXO2 | ID_1708 | 13 | 0 | 0.39 | 0.58 | 2.03 |
| P05164 | MPO | ID_319 | 13 | 0 | -0.74 | -0.56 | 2.02 |
| P07998 | RNASE1 | ID_358 | 13 | 0 | -1.45 | -1.34 | 2.02 |
| Q01459 | CTBS | ID_899 | 13 | 0 | -0.03 | 0.06 | 2 |
| Q15257 | PTPA | ID_1072 | 13 | 0 | -0.01 | 0.09 | 2 |

Early down-regulation in ADM

| **PG.ProteinAccessions** | **GENE Names** | **SPOT** | **Profile** | **A_ADM** | **B_ADM** | **C_ADM** | **D_ADM** |
| --- | --- | --- | --- | --- | --- | --- | --- |
| P20591 | MX1 | ID_518 | 9 | 0 | -0.09 | -2.3 | -5.04 |
| P08779 | KRT16 | ID_380 | 9 | 0 | -0.16 | -2.93 | -4.35 |
| P05109 | S100A8 | ID_313 | 9 | 0 | -0.02 | -1.42 | -4.31 |
| P06702 | S100A9 | ID_336 | 9 | 0 | 0.28 | -0.94 | -3.64 |
| P29728 | OAS2 | ID_599 | 9 | 0 | -0.4 | -2.13 | -3.52 |
| Q5K651 | SAMD9 | ID_1146 | 9 | 0 | -0.07 | -2.3 | -3.29 |
| P19971 | TYMP | ID_513 | 9 | 0 | -0.01 | -1.14 | -3.14 |
| P43490 | NAMPT | ID_701 | 10 | 0 | -0.13 | -2.24 | -3.04 |
| Q02487 | DSC2 | ID_911 | 9 | 0 | -0.43 | -1.95 | -3.01 |
| Q9HCS2 | CYP4F12 | ID_1591 | 9 | 0 | -0.15 | -0.91 | -3.01 |
| O95361 | TRIM16 | ID_207 | 9 | 0 | 0.04 | -0.93 | -2.99 |
| P29034 | S100A2 | ID_589 | 9 | 0 | -0.06 | -1.62 | -2.94 |
| P05161 | ISG15 | ID_318 | 10 | 0 | -0.19 | -2.74 | -2.74 |
| Q8IY21 | DDX60 | ID_1271 | 9 | 0 | -0.19 | -0.78 | -2.69 |
| Q9H190 | SDCBP2 | ID_1533 | 9 | 0 | 0.02 | -1.16 | -2.68 |
| Q9UIV8 | SERPINB13 | ID_1700 | 9 | 0 | 0.17 | -1.68 | -2.52 |
| P35250 | RFC2 | ID_643 | 9 | 0 | -0.18 | -1.01 | -2.4 |
| Q01469 | FABP5 | ID_900 | 9 | 0 | 0.34 | -0.7 | -2.38 |
| O60218 | AKR1B10 | ID_119 | 9 | 0 | -0.03 | -1.44 | -2.35 |
| Q8N4X5 | AFAP1L2 | ID_1296 | 9 | 0 | -0.45 | -1.07 | -2.3 |
| P31151 | S100A7 | ID_618 | 9 | 0 | 0.09 | -0.64 | -2.28 |
| Q5T447 | HECTD3 | ID_1155 | 9 | 0 | -0.27 | -0.74 | -2.19 |
| O15217 | GSTA4 | ID_81 | 9 | 0 | -0.12 | -1.03 | -2.15 |
| P41227 | NAA10 | ID_680 | 9 | 0 | -0.1 | -0.79 | -2.14 |
| Q6P1M0 | SLC27A4 | ID_1178 | 0 | 0 | -0.3 | -1.5 | -2.14 |
| P37268 | FDFT1 | ID_663 | 9 | 0 | -0.17 | -1.49 | -2.11 |
| P42224 | STAT1 | ID_685 | 9 | 0 | -0.22 | -1.27 | -2.11 |
| O14929 | HAT1 | ID_63 | 9 | 0 | -0.25 | -0.78 | -2.1 |
| O95786 | DDX58 | ID_218 | 9 | 0 | -0.21 | -0.89 | -2.08 |
| Q86UK0 | ABCA12 | ID_1247 | 10 | 0 | -0.31 | -1.69 | -2.07 |
| Q15738 | NSDHL | ID_1090 | 9 | 0 | -0.18 | -1.26 | -2.05 |
| O14879 | IFIT3 | ID_62 | 9 | 0 | 0 | -0.57 | -2.03 |
| Q99732 | LITAF | ID_1455 | 9 | 0 | -0.01 | -0.57 | -2.01 |

Lately down-regulation in ADM

| **PG.ProteinAccessions** | **GENE Names** | **SPOT** | **Profile** | **A_ADM** | **B_ADM** | **C_ADM** | **D_ADM** |
| --- | --- | --- | --- | --- | --- | --- | --- |
| Q8WWM7 | ATXN2L | ID_1335 | 12 | 0 | -0.11 | -0.02 | -4.98 |
| P40937 | RFC5 | ID_676 | 12 | 0 | -0.24 | -0.19 | -4.28 |
| Q9NS69 | TOMM22 | ID_1615 | 12 | 0 | 0.47 | 0.27 | -4.28 |
| Q8IXJ6 | SIRT2 | ID_1268 | 12 | 0 | -1.2 | 1.19 | -4.25 |
| O75676 | RPS6KA4 | ID_168 | 12 | 0 | -0.17 | -0.54 | -3.59 |
| Q15648 | MED1 | ID_1088 | 12 | 0 | -0.06 | 0.54 | -3.35 |
| Q5VW32 | BROX | ID_1160 | 12 | 0 | -0.5 | -0.09 | -2.89 |
| O95867 | LY6G6C | ID_226 | 12 | 0 | 0.04 | -0.21 | -2.8 |
| Q03701 | CEBPZ | ID_922 | 12 | 0 | -0.53 | -0.85 | -2.64 |
| Q96BH1 | RNF25 | ID_1387 | 12 | 0 | -0.51 | -0.63 | -2.37 |
| Q9HCU5 | PREB | ID_1592 | 12 | 0 | 0.67 | 1.86 | -2.36 |
| P42126 | ECI1 | ID_684 | 12 | 0 | 0.08 | 0.24 | -2.34 |
| P18433 | PTPRA | ID_504 | 12 | 0 | 0.22 | 1.08 | -2.33 |
| Q86U38 | NOP9 | ID_1244 | 12 | 0 | -0.3 | -0.51 | -2.24 |
| O75881 | CYP7B1 | ID_176 | 12 | 0 | -0.06 | -0.42 | -2.23 |
| Q969Z0 | TBRG4 | ID_1379 | 12 | 0 | -0.02 | -0.21 | -2.22 |
| Q7Z434 | MAVS | ID_1230 | 12 | 0 | 0.02 | 0.34 | -2.15 |
| Q96MW5 | COG8 | ID_1424 | 12 | 0 | 0 | 0.01 | -2.15 |
| Q9Y696 | CLIC4 | ID_1790 | 12 | 0 | -0.18 | 0.45 | -2.13 |
| Q06265 | EXOSC9 | ID_935 | 12 | 0 | 0.23 | 0.1 | -2.09 |
| O15027 | SEC16A | ID_69 | 12 | 0 | -0.01 | 0.44 | -2.08 |
| O00182 | LGALS9 | ID_35 | 12 | 0 | 0.22 | 0.12 | -2.07 |
| Q6ZV73 | FGD6 | ID_1207 | 12 | 0 | 0.13 | 1.64 | -2.07 |
| Q9NPQ8 | RIC8A | ID_1601 | 12 | 0 | -0.24 | -0.15 | -2.06 |
| Q8IZT6 | ASPM | ID_1280 | 12 | 0 | -0.08 | 1.01 | -2.04 |

Early up-regulation in BJS

| **PG.ProteinAccessions** | **GENE Names** | **SPOT** | **Profile** | **A_ADM** | **B_ADM** | **C_ADM** | **D_ADM** |
| --- | --- | --- | --- | --- | --- | --- | --- |
| P29317 | EPHA2 | ID_381 | 16 | 0 | -0.32 | 5.12 | 7.9 |
| Q12959 | DLG1 | ID_641 | 16 | 0 | 0.01 | 3.53 | 6.53 |
| P42858 | HTT | ID_452 | 16 | 0 | -0.08 | 2.66 | 5.37 |
| O15067 | PFAS | ID_48 | 25 | 0 | 2.09 | 3.38 | 4.92 |
| Q4ZHG4 | FNDC1 | ID_756 | 25 | 0 | 1.33 | 2.19 | 4.52 |
| Q9HCU4 | CELSR2 | ID_1036 | 16 | 0 | 0.23 | 1.84 | 4.38 |
| Q5D862 | FLG2 | ID_768 | 25 | 0 | 1.1 | 2.79 | 4.37 |
| Q8IUR6 | CREBRF | ID_841 | 22 | 0 | 1.06 | 1.43 | 4.33 |
| O60911 | CTSV | ID_97 | 16 | 0 | 0.57 | 1.82 | 4.05 |
| Q6NUJ1 | PSAPL1 | ID_793 | 25 | 0 | 0.68 | 2.2 | 3.72 |
| Q8IW75 | SERPINA12 | ID_844 | 25 | 0 | 1.1 | 2.21 | 3.54 |
| Q8WWT9 | SLC13A3 | ID_885 | 16 | 0 | -0.38 | 1.08 | 3.44 |
| O95864 | FADS2 | ID_160 | 25 | 0 | 1.1 | 2.48 | 3.39 |
| Q08345 | DDR1 | ID_629 | 16 | 0 | 0.53 | 1.53 | 3.36 |
| Q9UBW7 | ZMYM2 | ID_1094 | 25 | 0 | 1.32 | 2.28 | 3.27 |
| Q15417 | CNN3 | ID_713 | 16 | 0 | 0.16 | 1.95 | 3.2 |
| Q96RL7 | VPS13A | ID_951 | 22 | 0 | 1.78 | 2.17 | 3.09 |
| P78563 | ADARB1 | ID_583 | 16 | 0 | -0.07 | 0.92 | 3.04 |
| O75915 | ARL6IP5 | ID_126 | 16 | 0 | -0.19 | 1.11 | 2.95 |
| Q6ZQQ6 | WDR87 | ID_808 | 16 | 0 | 0.52 | 1.6 | 2.94 |
| P42771 | CDKN2A | ID_450 | 22 | 0 | 1.84 | 1.72 | 2.9 |
| O95832 | CLDN1 | ID_158 | 16 | 0 | -0.57 | 0.85 | 2.89 |
| Q9BX66 | SORBS1 | ID_987 | 25 | 0 | 0.94 | 1.88 | 2.84 |
| Q9NZH6 | IL37 | ID_1072 | 25 | 0 | 0.83 | 1.53 | 2.76 |
| O60240 | PLIN1 | ID_83 | 16 | 0 | 0.22 | 1.72 | 2.73 |
| P15090 | FABP4 | ID_304 | 25 | 0 | 0.81 | 1.91 | 2.69 |
| P20908 | COL5A1 | ID_338 | 16 | 0 | 0.17 | 1.12 | 2.67 |
| Q15828 | CST6 | ID_722 | 25 | 0 | 0.57 | 1.67 | 2.66 |
| P07951 | TPM2 | ID_226 | 16 | 0 | 0.42 | 0.91 | 2.65 |
| Q96BJ3 | AIDA | ID_919 | 25 | 0 | 1 | 1.64 | 2.61 |
| Q15057 | ACAP2 | ID_701 | 25 | 0 | 0.57 | 1.3 | 2.59 |
| P07098 | LIPF | ID_216 | 16 | 0 | -0.97 | 1.03 | 2.56 |
| P02462 | COL4A1 | ID_184 | 16 | 0 | -0.15 | 1.31 | 2.55 |
| P02753 | RBP4 | ID_190 | 22 | 0 | 0.89 | 0.93 | 2.51 |
| Q15121 | PEA15 | ID_704 | 16 | 0 | 0.29 | 0.75 | 2.51 |
| P05452 | CLEC3B | ID_207 | 16 | 0 | 0.19 | 1.31 | 2.5 |
| Q8N7A1 | KLHDC1 | ID_870 | 22 | 0 | 1.08 | 1.27 | 2.5 |
| P25311 | AZGP1 | ID_362 | 16 | 0 | 0.24 | 1.14 | 2.49 |
| O60888 | CUTA | ID_96 | 22 | 0 | 0.62 | 0.81 | 2.47 |
| Q02952 | AKAP12 | ID_606 | 16 | 0 | -0.13 | 0.88 | 2.44 |
| Q96FV2 | SCRN2 | ID_929 | 16 | 0 | -0.01 | 1.08 | 2.39 |
| P02724 | GYPA | ID_188 | 16 | 0 | -0.6 | 0.51 | 2.36 |
| Q4UJ75 | ANKRD20A4;ANKRD20A8P;ANKRD20A2;ANKRD20A1;ANKRD20A3 | ID_754 | 25 | 0 | 0.61 | 1.43 | 2.36 |
| Q7Z794 | KRT77 | ID_831 | 25 | 0 | 0.55 | 1.5 | 2.33 |
| O00142 | TK2 | ID_9 | 16 | 0 | 0.36 | 0.86 | 2.29 |
| P02461 | COL3A1 | ID_183 | 16 | 0 | -0.02 | 1.33 | 2.26 |
| P51888 | PRELP | ID_502 | 16 | 0 | 0.39 | 1.15 | 2.22 |
| Q9Y646 | CPQ | ID_1170 | 25 | 0 | 0.47 | 1.57 | 2.21 |
| Q9NR45 | NANS | ID_1048 | 16 | 0 | -0.15 | 1.39 | 2.19 |
| P78559 | MAP1A | ID_582 | 16 | 0 | 0.35 | 0.81 | 2.18 |
| O14717 | TRDMT1 | ID_32 | 22 | 0 | 0.85 | 0.98 | 2.16 |
| P04040 | CAT | ID_194 | 16 | 0 | 0.32 | 0.67 | 2.13 |
| P60033 | CD81 | ID_534 | 16 | 0 | 0.04 | 0.82 | 2.13 |
| Q96PE2 | ARHGEF17 | ID_947 | 25 | 0 | 0.72 | 1.2 | 2.13 |
| P35908 | KRT2 | ID_425 | 25 | 0 | 0.69 | 1.5 | 2.12 |
| P51688 | SGSH | ID_499 | 25 | 0 | 0.56 | 1.02 | 2.12 |
| P00746 | CFD | ID_173 | 25 | 0 | 0.42 | 1.18 | 2.1 |
| P35080 | PFN2 | ID_413 | 22 | 0 | 0.74 | 1.05 | 2.1 |
| Q8IZU8 | DSEL | ID_855 | 22 | 0 | 0.65 | 0.85 | 2.1 |
| Q13867 | BLMH | ID_672 | 25 | 0 | 0.56 | 1.07 | 2.09 |
| Q92820 | GGH | ID_903 | 16 | 0 | 0.28 | 0.65 | 2.09 |
| P26927 | MST1 | ID_371 | 25 | 0 | 0.66 | 1.02 | 2.08 |
| Q9Y337 | KLK5 | ID_1154 | 16 | 0 | 0.46 | 0.87 | 2.07 |
| Q12860 | CNTN1 | ID_638 | 16 | 0 | 0.28 | 0.92 | 2.05 |
| Q2VWP7 | PRTG | ID_750 | 16 | 0 | -0.12 | 1.44 | 2.03 |
| Q01546 | KRT76 | ID_598 | 16 | 0 | 0.08 | 1.21 | 2.02 |
| Q5T750 | XP32 | ID_776 | 25 | 0 | 0.65 | 1.29 | 2.02 |
| Q5XKE5 | KRT79 | ID_778 | 25 | 0 | 0.79 | 1.19 | 2.01 |
| A0A075B7D8 | IGHV3OR15-7 | ID_1 | 22 | 0 | 0.98 | 0.79 | 2 |
| P35625 | TIMP3 | ID_422 | 16 | 0 | 0.21 | 0.79 | 2 |

Lately up-regulation in BJS

| **PG.ProteinAccessions** | **GENE Names** | **SPOT** | **Profile** | **A_ADM** | **B_ADM** | **C_ADM** | **D_ADM** |
| --- | --- | --- | --- | --- | --- | --- | --- |
| Q6P587 | FAHD1 | ID_797 | 13 | 0 | 0.34 | 0.63 | 8.84 |
| Q16352 | INA | ID_725 | 13 | 0 | 0.3 | -0.3 | 7.46 |
| Q10472 | GALNT1 | ID_635 | 13 | 0 | 0.25 | 0.52 | 6.98 |
| O14981 | BTAF1 | ID_44 | 13 | 0 | 1.58 | -0.56 | 4.88 |
| Q9H2K8 | TAOK3 | ID_1013 | 13 | 0 | -1.07 | -0.26 | 4.04 |
| O60216 | RAD21 | ID_81 | 13 | 0 | 0.06 | 0.45 | 3.65 |
| Q69YN4 | VIRMA | ID_785 | 13 | 0 | 0.77 | 0.13 | 3.41 |
| P51553 | IDH3G | ID_493 | 13 | 0 | 0.18 | 0.25 | 3.37 |
| Q6ZS81 | WDFY4 | ID_810 | 13 | 0 | -0.76 | 0.45 | 3.33 |
| Q96BY7 | ATG2B | ID_920 | 13 | 0 | 0.11 | -0.44 | 3.25 |
| A0A075B6S2 | IGKV2D-29;IGKV2-29 | ID_0 | 13 | 0 | 0.29 | 0.19 | 3.23 |
| Q92917 | GPKOW | ID_906 | 13 | 0 | 0.71 | 0.82 | 3.17 |
| Q15036 | SNX17 | ID_700 | 13 | 0 | 0.27 | -0.1 | 3.05 |
| Q96T37 | RBM15 | ID_957 | 13 | 0 | 0.47 | 0.94 | 3.02 |
| P16083 | NQO2 | ID_308 | 13 | 0 | 0.13 | 0.7 | 2.98 |
| P13716 | ALAD | ID_294 | 13 | 0 | 0.18 | 0.37 | 2.94 |
| Q92615 | LARP4B | ID_897 | 13 | 0 | -1.45 | -1.04 | 2.94 |
| O75094 | SLIT3 | ID_99 | 13 | 0 | -0.06 | 0.01 | 2.9 |
| O75312 | ZPR1 | ID_104 | 13 | 0 | 0.32 | -0.29 | 2.82 |
| Q7LBC6 | KDM3B | ID_821 | 13 | 0 | -0.19 | -0.56 | 2.78 |
| Q14671 | PUM1 | ID_689 | 13 | 0 | 0.16 | -0.18 | 2.74 |
| Q92522 | H1-10 | ID_893 | 13 | 0 | -0.1 | 0.31 | 2.74 |
| Q14956 | GPNMB | ID_694 | 13 | 0 | 0.02 | 0.24 | 2.72 |
| Q9UHA4 | LAMTOR3 | ID_1106 | 13 | 0 | -0.01 | 0.16 | 2.7 |
| Q8NBJ5 | COLGALT1 | ID_873 | 13 | 0 | -0.05 | 0.21 | 2.68 |
| Q8N2G8 | GHDC | ID_860 | 13 | 0 | 0.51 | 0.68 | 2.67 |
| Q05086 | UBE3A | ID_615 | 13 | 0 | -0.05 | -0.15 | 2.6 |
| Q92625 | ANKS1A | ID_898 | 13 | 0 | -1.1 | -1.55 | 2.59 |
| P28072 | PSMB6 | ID_377 | 13 | 0 | 0.22 | -0.15 | 2.53 |
| Q8N0X4 | CLYBL | ID_856 | 13 | 0 | 0.35 | -1.25 | 2.53 |
| P22352 | GPX3 | ID_352 | 13 | 0 | 0.13 | 0.31 | 2.52 |
| P42773 | CDKN2C | ID_451 | 13 | 0 | -0.07 | 0.26 | 2.52 |
| O95352 | ATG7 | ID_150 | 13 | 0 | 0.2 | 0.19 | 2.49 |
| P20036 | HLA-DPA1 | ID_333 | 13 | 0 | 0.06 | -0.05 | 2.43 |
| P35914 | HMGCL | ID_426 | 13 | 0 | 0.32 | 0.65 | 2.41 |
| Q14CZ7 | FASTKD3 | ID_698 | 13 | 0 | -0.21 | 0.36 | 2.38 |
| P35443 | THBS4 | ID_417 | 13 | 0 | -0.18 | 0.38 | 2.31 |
| Q9BX68 | HINT2 | ID_988 | 13 | 0 | 0.45 | 0.65 | 2.31 |
| Q9BZA7 | PCDH11X | ID_999 | 13 | 0 | 0.39 | 0.29 | 2.3 |
| O60500 | NPHS1 | ID_86 | 13 | 0 | -0.19 | 0.1 | 2.28 |
| P10643 | C7 | ID_269 | 13 | 0 | 0.18 | 0.61 | 2.28 |
| Q9NPJ6 | MED4 | ID_1043 | 13 | 0 | 0.31 | 0.22 | 2.28 |
| Q9P2X3 | IMPACT | ID_1089 | 13 | 0 | 0.27 | 0.4 | 2.28 |
| P61204 | ARF3;ARF1 | ID_544 | 13 | 0 | -0.15 | 0.47 | 2.27 |
| Q9Y3B4 | SF3B6 | ID_1157 | 13 | 0 | -0.01 | 0.29 | 2.25 |
| Q9UEW8 | STK39 | ID_1098 | 13 | 0 | 0.38 | 0.5 | 2.22 |
| Q99471 | PFDN5 | ID_961 | 13 | 0 | -0.11 | 0.06 | 2.21 |
| Q9HCG8 | CWC22 | ID_1035 | 13 | 0 | 0.07 | -0.2 | 2.2 |
| Q562E7 | WDR81 | ID_764 | 13 | 0 | 0.12 | 0.08 | 2.19 |
| P09105 | HBQ1 | ID_245 | 13 | 0 | -0.39 | 0.29 | 2.15 |
| P32970 | CD70 | ID_405 | 13 | 0 | -0.19 | 0.26 | 2.14 |
| Q8N755 | SLC66A3 | ID_869 | 13 | 0 | 0.15 | 0.43 | 2.13 |
| Q9HBI1 | PARVB | ID_1029 | 13 | 0 | 0.27 | 0.48 | 2.12 |
| P02647 | APOA1 | ID_186 | 13 | 0 | -0.06 | -0.14 | 2.1 |
| P22033 | MMUT | ID_349 | 13 | 0 | 0.1 | 0.36 | 2.08 |
| Q4V328 | GRIPAP1 | ID_755 | 13 | 0 | 0.37 | -0.01 | 2.08 |
| O00479 | HMGN4 | ID_20 | 13 | 0 | 0.6 | 0.18 | 2.07 |
| Q13769 | THOC5 | ID_668 | 13 | 0 | 0.29 | 0.02 | 2.07 |
| Q9UBR2 | CTSZ | ID_1092 | 13 | 0 | 0.42 | 0.45 | 2.07 |
| O60925 | PFDN1 | ID_98 | 13 | 0 | 0.27 | 0.22 | 2.06 |
| Q13094 | LCP2 | ID_643 | 13 | 0 | -0.12 | -0.2 | 2.06 |
| P0CG38 | POTEI | ID_257 | 13 | 0 | 0.37 | 0.15 | 2.04 |
| P37235 | HPCAL1 | ID_432 | 13 | 0 | -0.43 | 0.18 | 2.03 |
| P30041 | PRDX6 | ID_389 | 13 | 0 | 0.06 | 0.13 | 2.01 |

Early down-regulation in BJS

| **PG.ProteinAccessions** | **GENE Names** | **SPOT** | **Profile** | **A_ADM** | **B_ADM** | **C_ADM** | **D_ADM** |
| --- | --- | --- | --- | --- | --- | --- | --- |
| P29508 | SERPINB3 | ID_384 | 0 | 0 | -0.89 | -2.11 | -4.2 |
| P08779 | KRT16 | ID_241 | 9 | 0 | -0.54 | -2.55 | -3.96 |
| P20591 | MX1 | ID_336 | 9 | 0 | -0.45 | -1.48 | -3.83 |
| P48594 | SERPINB4 | ID_474 | 0 | 0 | -1.13 | -2.51 | -3.53 |
| P05109 | S100A8 | ID_201 | 9 | 0 | -0.12 | -1.25 | -3.41 |
| P29728 | OAS2 | ID_387 | 9 | 0 | -0.2 | -1.28 | -3.4 |
| Q5JRX3 | PITRM1 | ID_769 | 0 | 0 | -0.94 | -2.22 | -3.05 |
| P06702 | S100A9 | ID_213 | 9 | 0 | -0.22 | -1.14 | -3.02 |
| Q6NT55 | CYP4F22 | ID_792 | 0 | 0 | -0.64 | -2.17 | -2.67 |
| O60218 | AKR1B10 | ID_82 | 9 | 0 | -0.11 | -1.83 | -2.65 |
| P35916 | FLT4 | ID_427 | 0 | 0 | -0.66 | -1.55 | -2.6 |
| P19971 | TYMP | ID_332 | 9 | 0 | 0.01 | -0.64 | -2.43 |
| P08195 | SLC3A2 | ID_231 | 9 | 0 | 0.11 | -0.79 | -2.35 |
| Q02487 | DSC2 | ID_603 | 9 | 0 | -0.21 | -1.57 | -2.3 |
| P43490 | NAMPT | ID_456 | 10 | 0 | -0.39 | -1.89 | -2.25 |
| P29034 | S100A2 | ID_379 | 9 | 0 | -0.01 | -1.39 | -2.14 |
| Q8IY21 | DDX60 | ID_850 | 9 | 0 | 0.03 | -0.62 | -2.14 |

Lately down-regulation in BJS

| **PG.ProteinAccessions** | **GENE Names** | **SPOT** | **Profile** | **A_ADM** | **B_ADM** | **C_ADM** | **D_ADM** |
| --- | --- | --- | --- | --- | --- | --- | --- |
| P22528 | SPRR1B | ID_354 | 12 | 0 | -0.24 | -0.9 | -3.38 |
| Q96TA2 | YME1L1 | ID_958 | 12 | 0 | 0.47 | 0.07 | -3.15 |
| P42126 | ECI1 | ID_444 | 12 | 0 | 0.05 | 0.58 | -2.77 |
| O60884 | DNAJA2 | ID_95 | 12 | 0 | 0.3 | -0.36 | -2.56 |
| Q01130 | SRSF2 | ID_595 | 12 | 0 | -0.62 | -0.43 | -2.3 |
| Q9H7C9 | AAMDC | ID_1018 | 12 | 0 | -0.68 | -0.21 | -2.19 |
| O14639 | ABLIM1 | ID_30 | 12 | 0 | 0.43 | 1.43 | -2.15 |

**Table S2** Pearson correlation between DEPs and PASI in validation research.

P-value

| PG.ProteinId | PG.GeneId | PG.ProteinDescription | P-value-AWJ | P-value-ADM | P-value-BJS |
| --- | --- | --- | --- | --- | --- |
| A8TX70 | COL6A5 | Collagen alpha-5(VI) chain | 0.693672 | 0.004513 | 0.485931 |
| P00734 | F2 | Prothrombin | 0.794035 | 0.504699 | 0.192197 |
| P02461 | COL3A1 | Collagen alpha-1(III) chain | 0.555422 | 0.122211 | 0.869309 |
| P02760 | AMBP | Protein AMBP | 0.721802 | 0.378906 | 0.969388 |
| P02765 | AHSG | Alpha-2-HS-glycoprotein | 0.671383 | 0.203229 | 0.641489 |
| P02768 | ALB | Albumin | 0.801137 | 0.216353 | 0.886717 |
| P02774 | GC | Vitamin D-binding protein | 0.92034 | 0.480988 | 0.426892 |
| P02786 | TFRC | Transferrin receptor protein 1 | 0.63259 | 0.015887 | 0.001281 |
| P02787 | TF | Serotransferrin | 0.875749 | 0.246988 | 0.501887 |
| P04179 | SOD2 | Superoxide dismutase [Mn], mitochondrial | 0.828865 | 0.077075 | 0.001929 |
| P05089 | ARG1 | Arginase-1 | 0.780089 | 0.723509 | 0.879662 |
| P05109 | S100A8 | Protein S100-A8 | 0.298155 | 0.131704 | 0.001436 |
| P06702 | S100A9 | Protein S100-A9 | 0.321256 | 0.127121 | 0.001889 |
| P07996 | THBS1 | Thrombospondin-1 | 0.310883 | 8.78E-05 | 0.123302 |
| P08238 | HSP90AB1 | Heat shock protein HSP 90-beta | 0.275087 | 0.052379 | 0.001665 |
| P08697 | SERPINF2 | Alpha-2-antiplasmin | 0.700538 | 0.970002 | 0.065529 |
| P12268 | IMPDH2 | Inosine-5'-monophosphate dehydrogenase 2 | 0.512138 | 0.005225 | 0.006113 |
| P17931 | LGALS3 | Galectin-3 | 0.021132 | 0.000878 | 0.102156 |
| P19971 | TYMP | Thymidine phosphorylase | 0.082278 | 0.20719 | 0.009214 |
| P20908 | COL5A1 | Collagen alpha-1(V) chain | 0.446863 | 0.424641 | 0.40564 |
| P25787 | PSMA2 | Proteasome subunit alpha type-2 | 0.743679 | 0.382128 | 0.03668 |
| P31151 | S100A7 | Protein S100-A7 | 0.648224 | 0.29952 | 0.002248 |
| P35908 | KRT2 | Keratin, type II cytoskeletal 2 epidermal | 0.709562 | 0.013006 | 0.001376 |
| P36955 | SERPINF1 | Pigment epithelium-derived factor | 0.190785 | 0.490258 | 0.488999 |
| P42224 | STAT1 | Signal transducer and activator of transcription 1-alpha/beta | 0.0744 | 0.19715 | 0.004237 |
| Q15746 | MYLK | Myosin light chain kinase, smooth muscle | 0.769926 | 0.217228 | 0.255538 |
| Q96FX8 | PERP | p53 apoptosis effector related to PMP-22 | 0.326927 | 0.891447 | 0.75671 |

correlation coefficient

| PG.ProteinId | PG.GeneId | PG.ProteinDescription | correlation coefficient-AWJ | correlation coefficient-ADM | correlation coefficient-BJS |
| --- | --- | --- | --- | --- | --- |
| A8TX70 | COL6A5 | Collagen alpha-5(VI) chain | 0.093926 | -0.60731 | -0.16538 |
| P00734 | F2 | Prothrombin | 0.062335 | 0.158426 | 0.304226 |
| P02461 | COL3A1 | Collagen alpha-1(III) chain | -0.14023 | 0.357081 | 0.039308 |
| P02760 | AMBP | Protein AMBP | -0.08494 | -0.20798 | 0.009171 |
| P02765 | AHSG | Alpha-2-HS-glycoprotein | -0.10113 | -0.29717 | 0.110936 |
| P02768 | ALB | Albumin | -0.06014 | -0.28911 | 0.034038 |
| P02774 | GC | Vitamin D-binding protein | -0.0239 | -0.16723 | 0.188181 |
| P02786 | TFRC | Transferrin receptor protein 1 | 0.113887 | 0.531457 | 0.668196 |
| P02787 | TF | Serotransferrin | -0.03736 | -0.27145 | 0.15946 |
| P04179 | SOD2 | Superoxide dismutase [Mn], mitochondrial | 0.051626 | 0.404273 | 0.649776 |
| P05089 | ARG1 | Arginase-1 | 0.066654 | -0.0844 | 0.036172 |
| P05109 | S100A8 | Protein S100-A8 | 0.24484 | 0.348838 | 0.663192 |
| P06702 | S100A9 | Protein S100-A9 | 0.233749 | 0.352762 | 0.650755 |
| P07996 | THBS1 | Thrombospondin-1 | -0.23867 | 0.764128 | 0.356111 |
| P08238 | HSP90AB1 | Heat shock protein HSP 90-beta | 0.256452 | 0.439717 | 0.656543 |
| P08697 | SERPINF2 | Alpha-2-antiplasmin | -0.09172 | 0.008988 | 0.419576 |
| P12268 | IMPDH2 | Inosine-5'-monophosphate dehydrogenase 2 | 0.155705 | 0.599347 | 0.590575 |
| P17931 | LGALS3 | Galectin-3 | -0.51158 | -0.68416 | -0.37614 |
| P19971 | TYMP | Thymidine phosphorylase | 0.397933 | 0.294703 | 0.566465 |
| P20908 | COL5A1 | Collagen alpha-1(V) chain | -0.1803 | 0.189082 | -0.19679 |
| P25787 | PSMA2 | Proteasome subunit alpha type-2 | 0.078029 | 0.206611 | 0.469648 |
| P31151 | S100A7 | Protein S100-A7 | 0.108713 | 0.244171 | 0.642565 |
| P35908 | KRT2 | Keratin, type II cytoskeletal 2 epidermal | -0.08884 | -0.54474 | -0.66508 |
| P36955 | SERPINF1 | Pigment epithelium-derived factor | 0.305148 | -0.16376 | -0.16423 |
| P42224 | STAT1 | Signal transducer and activator of transcription 1-alpha/beta | 0.407658 | 0.301027 | 0.61067 |
| Q15746 | MYLK | Myosin light chain kinase, smooth muscle | -0.06981 | 0.28858 | 0.266777 |
| Q96FX8 | PERP | p53 apoptosis effector related to PMP-22 | 0.2311 | -0.03261 | -0.07614 |
