# Supplementary material for: IL-17A and TNF-α inhibitors induce multiple molecular changes in psoriasis
Source: Front Immunol. 2022 Nov 22;13:1015182. doi: 10.3389/fimmu.2022.1015182 (PMC9723344; doi:10.3389/fimmu.2022.1015182)
Supplement: Supplementary file 7 [file Table_2.docx]

**Table S2** Pearson correlation between DEPs and PASI in validation research.

P-value

| PG.ProteinId | PG.GeneId | PG.ProteinDescription | P-value-AWJ | P-value-ADM | P-value-BJS |
| --- | --- | --- | --- | --- | --- |
| A8TX70 | COL6A5 | Collagen alpha-5(VI) chain | 0.693672 | 0.004513 | 0.485931 |
| P00734 | F2 | Prothrombin | 0.794035 | 0.504699 | 0.192197 |
| P02461 | COL3A1 | Collagen alpha-1(III) chain | 0.555422 | 0.122211 | 0.869309 |
| P02760 | AMBP | Protein AMBP | 0.721802 | 0.378906 | 0.969388 |
| P02765 | AHSG | Alpha-2-HS-glycoprotein | 0.671383 | 0.203229 | 0.641489 |
| P02768 | ALB | Albumin | 0.801137 | 0.216353 | 0.886717 |
| P02774 | GC | Vitamin D-binding protein | 0.92034 | 0.480988 | 0.426892 |
| P02786 | TFRC | Transferrin receptor protein 1 | 0.63259 | 0.015887 | 0.001281 |
| P02787 | TF | Serotransferrin | 0.875749 | 0.246988 | 0.501887 |
| P04179 | SOD2 | Superoxide dismutase [Mn], mitochondrial | 0.828865 | 0.077075 | 0.001929 |
| P05089 | ARG1 | Arginase-1 | 0.780089 | 0.723509 | 0.879662 |
| P05109 | S100A8 | Protein S100-A8 | 0.298155 | 0.131704 | 0.001436 |
| P06702 | S100A9 | Protein S100-A9 | 0.321256 | 0.127121 | 0.001889 |
| P07996 | THBS1 | Thrombospondin-1 | 0.310883 | 8.78E-05 | 0.123302 |
| P08238 | HSP90AB1 | Heat shock protein HSP 90-beta | 0.275087 | 0.052379 | 0.001665 |
| P08697 | SERPINF2 | Alpha-2-antiplasmin | 0.700538 | 0.970002 | 0.065529 |
| P12268 | IMPDH2 | Inosine-5'-monophosphate dehydrogenase 2 | 0.512138 | 0.005225 | 0.006113 |
| P17931 | LGALS3 | Galectin-3 | 0.021132 | 0.000878 | 0.102156 |
| P19971 | TYMP | Thymidine phosphorylase | 0.082278 | 0.20719 | 0.009214 |
| P20908 | COL5A1 | Collagen alpha-1(V) chain | 0.446863 | 0.424641 | 0.40564 |
| P25787 | PSMA2 | Proteasome subunit alpha type-2 | 0.743679 | 0.382128 | 0.03668 |
| P31151 | S100A7 | Protein S100-A7 | 0.648224 | 0.29952 | 0.002248 |
| P35908 | KRT2 | Keratin, type II cytoskeletal 2 epidermal | 0.709562 | 0.013006 | 0.001376 |
| P36955 | SERPINF1 | Pigment epithelium-derived factor | 0.190785 | 0.490258 | 0.488999 |
| P42224 | STAT1 | Signal transducer and activator of transcription 1-alpha/beta | 0.0744 | 0.19715 | 0.004237 |
| Q15746 | MYLK | Myosin light chain kinase, smooth muscle | 0.769926 | 0.217228 | 0.255538 |
| Q96FX8 | PERP | p53 apoptosis effector related to PMP-22 | 0.326927 | 0.891447 | 0.75671 |

correlation coefficient

| PG.ProteinId | PG.GeneId | PG.ProteinDescription | correlation coefficient-AWJ | correlation coefficient-ADM | correlation coefficient-BJS |
| --- | --- | --- | --- | --- | --- |
| A8TX70 | COL6A5 | Collagen alpha-5(VI) chain | 0.093926 | -0.60731 | -0.16538 |
| P00734 | F2 | Prothrombin | 0.062335 | 0.158426 | 0.304226 |
| P02461 | COL3A1 | Collagen alpha-1(III) chain | -0.14023 | 0.357081 | 0.039308 |
| P02760 | AMBP | Protein AMBP | -0.08494 | -0.20798 | 0.009171 |
| P02765 | AHSG | Alpha-2-HS-glycoprotein | -0.10113 | -0.29717 | 0.110936 |
| P02768 | ALB | Albumin | -0.06014 | -0.28911 | 0.034038 |
| P02774 | GC | Vitamin D-binding protein | -0.0239 | -0.16723 | 0.188181 |
| P02786 | TFRC | Transferrin receptor protein 1 | 0.113887 | 0.531457 | 0.668196 |
| P02787 | TF | Serotransferrin | -0.03736 | -0.27145 | 0.15946 |
| P04179 | SOD2 | Superoxide dismutase [Mn], mitochondrial | 0.051626 | 0.404273 | 0.649776 |
| P05089 | ARG1 | Arginase-1 | 0.066654 | -0.0844 | 0.036172 |
| P05109 | S100A8 | Protein S100-A8 | 0.24484 | 0.348838 | 0.663192 |
| P06702 | S100A9 | Protein S100-A9 | 0.233749 | 0.352762 | 0.650755 |
| P07996 | THBS1 | Thrombospondin-1 | -0.23867 | 0.764128 | 0.356111 |
| P08238 | HSP90AB1 | Heat shock protein HSP 90-beta | 0.256452 | 0.439717 | 0.656543 |
| P08697 | SERPINF2 | Alpha-2-antiplasmin | -0.09172 | 0.008988 | 0.419576 |
| P12268 | IMPDH2 | Inosine-5'-monophosphate dehydrogenase 2 | 0.155705 | 0.599347 | 0.590575 |
| P17931 | LGALS3 | Galectin-3 | -0.51158 | -0.68416 | -0.37614 |
| P19971 | TYMP | Thymidine phosphorylase | 0.397933 | 0.294703 | 0.566465 |
| P20908 | COL5A1 | Collagen alpha-1(V) chain | -0.1803 | 0.189082 | -0.19679 |
| P25787 | PSMA2 | Proteasome subunit alpha type-2 | 0.078029 | 0.206611 | 0.469648 |
| P31151 | S100A7 | Protein S100-A7 | 0.108713 | 0.244171 | 0.642565 |
| P35908 | KRT2 | Keratin, type II cytoskeletal 2 epidermal | -0.08884 | -0.54474 | -0.66508 |
| P36955 | SERPINF1 | Pigment epithelium-derived factor | 0.305148 | -0.16376 | -0.16423 |
| P42224 | STAT1 | Signal transducer and activator of transcription 1-alpha/beta | 0.407658 | 0.301027 | 0.61067 |
| Q15746 | MYLK | Myosin light chain kinase, smooth muscle | -0.06981 | 0.28858 | 0.266777 |
| Q96FX8 | PERP | p53 apoptosis effector related to PMP-22 | 0.2311 | -0.03261 | -0.07614 |
